# Evaluation of race-free eGFR equations in individuals of different ethnicity

**DOI:** 10.1080/08037051.2025.2533456

**Published:** 2025-07-15

**Authors:** De-Wei An, Gontse G. Mokwatsi, Dong-Yan Zhang, Dries S. Martens, Yu-Ling Yu, Babangida S. Chori, Augustine N. Odili, Ruan Kruger, Lebo F. Gafane-Matemane, Justyna Siwy, Agnieszka Latosinska, Harald Mischak, Catharina MC Mels, Aletta E. Schutte, Jean-René M’Buyamba-Kabangu, Tim S. Nawrot, Yan Li, Jan A. Staessen

**Affiliations:** a Department of Cardiovascular Medicine, Shanghai Key Laboratory of Hypertension, Shanghai Institute of Hypertension, State Key Laboratory of Medical Genomics, National Research Centre for Translational Medicine, Ruijin Hospital, Shanghai Jiaotong University School of Medicine, Shanghai, China; b Non-Profit Research Alliance for the Promotion of Preventive Medicine, Mechelen, Belgium; c Research Unit Environment and Health, KU Leuven Department of Public Health and Primary Care, University of Leuven, Leuven, Belgium; d Centre for Environmental Sciences, Hasselt University, Diepenbeek, Belgium; e Hypertension in Africa Research Team (HART), North-West University, Potchefstroom, South Africa; f SAMRC Extramural Unit for Hypertension and Cardiovascular Disease, North-West University, Potchefstroom, South Africa; g Circulatory Health Research Laboratory, College of Health Sciences, University of Abuja, Gwagwalada, Nigeria; h Mosaiques-Diagnostics GmbH, Hannover, Germany; i School of Population Health, University of New South Wales, The George Institute for Global Health; j Hypertension Unit, Department of Internal Medicine, University of Kinshasa, Hospital, Kinshasa, Democratic Republic of Congo; k The First Department of Cardiology, Interventional Electrocardiology and Hypertension, Jagiellonian University Medical College, Kraków, Poland; l Biomedical Sciences Group, University of Leuven, Leuven, Belgium

**Keywords:** Biomedical analytics, chronic kidney disease, estimated glomerular filtration rate, mortality, population science, race

## Abstract

**Background:**

Glomerular filtration rate (eGFR) derived from serum creatinine (eGFR_cr_), cystatin C (eGFR_cys_), or both (eGFR_cr-cys_) by race-free equations are recommended staging chronic kidney disease (CKD). The current study aimed to compare these race-free eGFR equations for screening for low-grade CKD in Blacks and non-Blacks and to evaluate their association with mortality.

**Methods:**

Race-free eGFR equations were evaluated in four studies with specific inclusion criteria based on the original research goals: African-PREDICT (341/380 healthy Black/White South Africans), FLEMENGHO (709 White community-dwelling Flemish), NHANES (1760/7931 Black and non-Black adult Americans), and 401 Black African patients hospitalised in Mbuji Mayi, Democratic Republic of Congo. The intraclass correlation coefficient and Bland and Altman statistics were used to assess consistency between eGFR equations and multivariable logistic or Cox regression to evaluate their association with mortality.

**Results:**

Intraindividual discordance between eGFRs was larger in Black than non-Black NHANES and African-PREDICT participants. In NHANES, eGFR_cr-cys_ was greater than eGFR_cr_, but smaller than eGFR_cys_, and replacing eGFR_cr-cys_ by eGFR_cr_ moved 25% Blacks and 15% non-Blacks to a higher (worse) eGFR KDIGO stage. In African-PREDICT and FLEMENGO, half of the measured creatinine clearance to eGFR ratios fell outside the expected 1.1–1.2 band. In NHANES, multivariable hazard ratios for total and cardiovascular mortality in relation to CKD grade were all lower than unity for grade-1 CKD and greater than unity for grade ≥3 (*p* < 0.0001) without any racial difference (0.11≤*p* ≤ 0.98). These NHANES findings were consistent, if CKD stage was replaced by eGFR and in subgroup analyses. Whereas eGFR_cys_ and eGFR_cr-cys_ refined models, eGFR_cr_ did not.

**Conclusions:**

The NHANES mortality outcomes support the use of eGFR_cys_ and eGFR_cr-cys_. However, large intraindividual variability between eGFR estimates may lead to KDIGO eGFR stage misclassification and calls for caution in the opportunistic or systematic screening for CKD in asymptomatic individuals with prevention as objective.

## PLAIN LANGUAGE SUMMARY

Glomerular filtration rate reflects the ability to remove excess water and waste by the kidney and can be estimated from serum creatinine (eGFR). However, serum creatinine is affected by muscle mass, dietary habits and other factors that cluster by race and lead to overestimation of eGFR and underestimation of the risk of kidney disease in Black individuals. Serum cystatin C is less affected by these confounders. Equations to compute eGFR from serum cystatin C or both serum creatinine and cystatin C without considering race are currently recommended. This study evaluated these race-free equations to compute eGFR in multi-ethnic study populations recruited in South Africa, Belgium, the Democratic Republic of the Congo and the United States. The new methods produce eGFR estimates, which predict mortality and have near perfect reproducibility in the population as a whole. However, this study also identified large intraindividual differences between eGFR estimates that may lead to misclassification of patients with regard to their renal function. These observations call for caution in the clinical application of equations to derive eGFR from serum markers and highlight the need for further research to optimise the prevention and management of chronic kidney disease.

## Background

The disease burden attributable to chronic kidney disease (CKD) is huge [1]. In 2017, CKD affected 698 million people with a worldwide annual fatality rate of 1.2 million deaths [1]. Estimated glomerular filtration rate (eGFR) is the tool par excellence for managing patients with established CKD [2]. Initially, eGFR equations included black race as cofactor [3,4], leading to an overestimation of eGFR and delaying CKD management in Blacks [5,6]. The Chronic Kidney Disease Epidemiology Collaboration (CKD-EPI) [7–9] and the European Kidney Function Consortium (EKFC) [10,11] proposed equations to derive eGFR from sex, age, serum creatinine (eGFR_cr_), serum cystatin C (eGFR_cys_), or both (eGFR_cr-cys_) without race factor. To develop these race-free eGFR equations, the CKD-EPI [8] and EKFC [11] consortia evaluated non-Black individuals from North America and Europe. In the CKD-EPI analysis [8], Black participants were African Americans, whilst in the EKFC study [11] Blacks were recruited in France, Ivory Coast and Congo.

The development of race-free eGFR equations addresses longstanding concerns that race, a social construct rather than a biological proxy, contributed to health inequalities by potentially delaying referral to nephrology and transplantation for Black patients whose eGFR was overestimated by race-inclusive models [12]. A key advantage of these new equations is their potential to prompt earlier, more equitable clinical action by providing unbiased eGFR estimates across diverse populations, especially when the additional biomarker cystatin C is used [13]. However, a notable disadvantage is that some initial race-free equations, such as the race-free CKD-EPI 2021 equation [8], have lower overall accuracy compared to older race-inclusive equations, especially in patients, who underwent kidney transplantation. This potential lowering of estimated eGFR for Black patients could, if not carefully managed, also raise concerns about their eligibility for beneficial medications. Nevertheless, a recent study in kidney transplant recipients, demonstrate that a newly developed race-free equation can achieve high accuracy across various subpopulations, including Black patients, and outperform the prior race-free CKD-EPI 2021 equation [14].

Although race-free eGFR equations [8,11] are currently recommended for clinical practice [2], their applicability is not well documented in assessing CKD risk at the population level [15–17]. The first objective of the current study was therefore to evaluate the concordance and accuracy of the race-free eGFR equations [8,11] in Black and non-Black individuals enrolled in three population studies, respectively conducted in South Africa [18]. Belgium [19] and the United States [20]. The second objective was to assess the association of mortality with CKD grade [2] as provided by race-free eGFRs in adults representative of the population in the United States [20] and in Black patients hospitalised in Mbuji Mayi, Democratic Republic of the Congo [21].

## Methods

**Figure 1.**
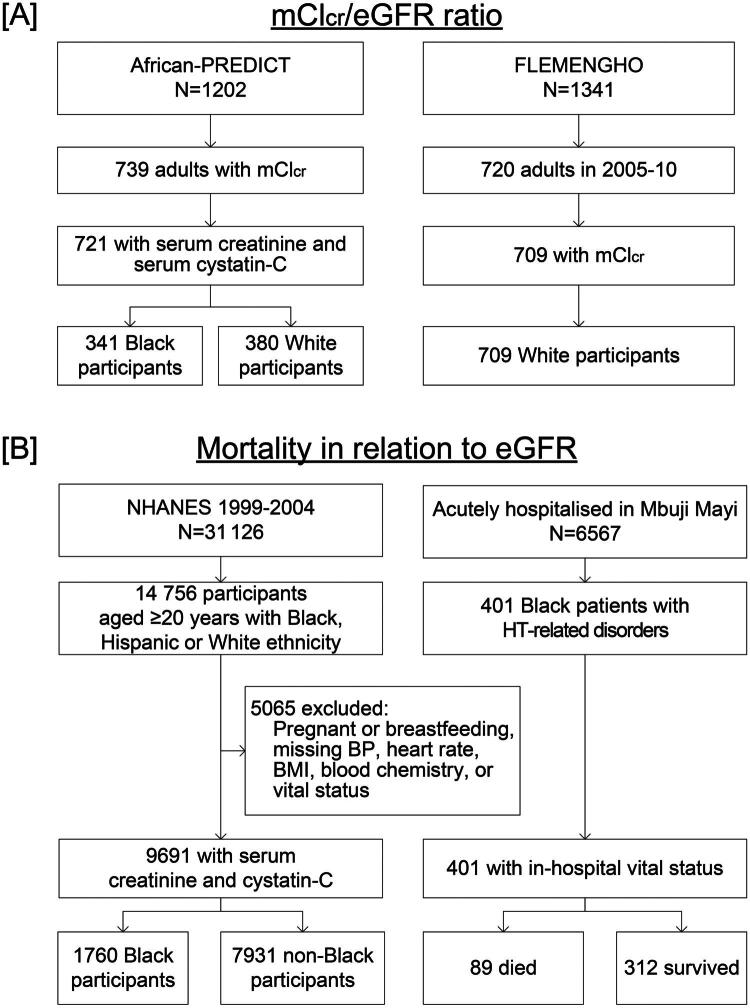
Flowchart showing the selection of study participants. The mCl_cr_/eGFR ratio was assessed in Black and White South Africans and in White Europeans, enrolled in African-PREDICT and FLEMENGHO, respectively (a). The association between mortality and eGFR derived from race-free equations was studied in Black and non-Black NHANES participants representative of the adult population of the United States and in Black hypertensive patients admitted to the emergency department of two hospitals in Mbuji Mayi, Democratic Republic of Congo (b). mCl_cr_: measured creatinine clearance; HT: hypertension; BP: blood pressure; BMI: body mass index.

This study combines cross-sectional and prospective data to serve its stated objectives (Figure 1). In cross-sectional studies, the primary outcome was the concordance and accuracy of the race-free CKD-EPI and EKFC equations when compared to eGFR_cr-cys_ and mCl_cr_ in Black and non-Black individuals. The secondary outcome of the cross-sectional analyses was the reclassification of individuals based on eGFR and the associated impact on CKD staging. In the prospective studies, the primary outcome was the association of mortality with eGFR. In secondary analyses, the consistency of the associations of mortality with eGFR was assessed across various subgroups and the extent to which eGFR refined the risk stratification over and beyond a base model including other risk factors.

### Study populations

All studies (Figure 1) complied with the Helsinki Declaration for research in humans [22]. Study protocols and sharing of anonymised data received approval from the competent Institutional Review Boards. Participants provided informed written consent. The African Prospective Study on the Early Detection and Identification of Cardiovascular Disease and Hypertension (African-PREDICT) [18] includes 1202 apparently healthy individuals, recruited from 2017 until 2021 from the community in Potchefstroom and surrounding areas in South Africa. Eligible participants were Black or White adults, aged 20–30 years, of whom none had hypertension, diabetes or a history of chronic disease, was HIV positive, or on treatment with antihypertensive, lipid-lowering or antidiabetic drugs. The Flemish Study of the Environment, Genes and Health Outcomes (FLEMENGHO) is a longitudinal family-based population study, conducted in Northern Belgium (Noordkempen) [19]. Recruitment started in 1985 and continued until 2008. All participants were White Europeans. From 2005 until 2010, 1208 participants were invited for follow-up at the examination centre located in the catchment area (Eksel, Belgium). Of 1055 surviving and non-institutionalised former participants still living in the catchment area, 828 (77.9%) renewed consent and 709 had all variables measured required for analysis.

The association of mortality with eGFR (Figure 1) was analysed using the baseline data from three 2-year cycles (1999–2004) of the National Health Nutrition Examination Survey (NHANES) in the United States [20]. Participants qualified for analysis, if they identified themselves as Black, Hispanic or White, if the required baseline information was available, including serum creatinine and serum cystatin C, and if vital status was known on 31 December 2019. Using probabilistic matching by means of a series of identifiers, the baseline data were linked with the cause of death as recorded in the National Death Index. The endpoints retrieved were all-cause mortality, cardiovascular mortality (ICD10 codes I00–I09, I11, I13, I20–I51, and I60–I69) and renal mortality (N00–N07, N17–N19, N25–N27). From 2001 until 2003 (Figure 1), 6567 patients were admitted to the Dipumba and Bonzola city hospitals in Mbuji Mayi, Kasai-Oriental Province, Democratic Republic of the Congo. The current analysis includes 401 patients consecutively hospitalised for hypertension or related complications. The reasons for admission were life-threatening cerebrovascular, cardiovascular or renal complications, symptomatic or severe hypertension associated or not with comorbid conditions, such as diabetes, or adverse reactions to antihypertensive drugs [21].

### Measurements

Seated blood pressure (BP) was the average of three readings in African-PREDICT and FLEMENGHO and of two to four readings in NHANES. In Mbuji Mayi, the supine BP was the average of two readings. Mean arterial pressure (MAP) was diastolic BP + 0.40 × (systolic BP – diastolic BP) [23]. African-PREDICT and FLEMENGHO participants collected exactly timed 24-h urine samples. Aliquoted urine samples were deep-frozen until analysed for creatinine. Cystatin C was measured on biobanked serum samples in African-PREDICT and NHANES. Creatinine was determined using Jaffe’s method with modifications in certified laboratories that applied isotope-dilution mass spectrometry for calibration [24] and serum cystatin C by the Dade Behring N Latex Cystatin C assay [25]. In African-PREDICT and FLEMENGHO, the measured creatinine clearance (mCl_cr_) was computed as 24-h urinary creatinine excretion/serum creatinine/1440 min. Chronic kidney disease was staged based on eGFR and using the 2024 KDIGO criteria [2]. Creatinine passes the glomerular sieve, but is also secreted by the proximal renal tubules [26]. Among adults studied under normal physiological conditions, the ratio of mCl_cr_ to the eGFR (mCl_cr_/eGFR) ranges from 1.1 to 1.2 [27]. The CKD-EPI 2021 [8] and EKFC 2023 [11] equations to compute race-free eGFR are given in Supplementary Tables 1–2. eGFR and mCl_cr_ were standardised to a body surface area of 1.73 m^2^.

### Statistical analysis

Statistical analyses were conducted using SAS, version 9.4 (SAS Institute, Cary, NC) and R, version 4.4.1 (R Core Team, Austria). Between-group comparisons were implemented by the large-sample z test and the χ^2^ statistic for continuously distributed and categorical variables, respectively. The agreement between various race-free eGFR equations at the group level was assessed by the intraclass correlation coefficient (ICC) [28] and the Bland and Altman method [29]. eGFR estimates were compared between research consortia (CKD-EPI 2021 *vs* EKFC 2023) and between racial groups. ICCs indicating perfect agreement are >0.80 [28]. Bias (Δ) in eGFR estimates was computed as (eGFR tested – eGFR reference) and plotted against the mean of eGFR tested and eGFR reference i.e. the consortium-specific eGFR_cr-cys_ [29]. Given the distribution of Δ, the 95% limits of agreement (95% LA) are mean Δ ± (1.96 × SD). Reclassification of individual participants by race was assessed in African-PREDICT and NHANES. Fisher exact test was used for within-group comparisons of proportions and the *κ* statistic for the overall consistency in the CKD classifications based on different eGFR formulations.

In the Mbuji Mayi cohort, the association of in-hospital mortality with race-free eGFR [8,11] was assessed by logistic regression unadjusted and adjusted for sex, age, body mass index (BMI), MAP, and antihypertensive treatment status. In NHANES, associations of mortality with CKD grade or race-free eGFR were evaluated by Cox regression, unadjusted, and with basic and extended adjustment, respectively accounting for sex, age, BMI and MAP, and next also for smoking, educational attainment and the poverty index. CKD stage was coded by the deviation-of-mean coding [30], which does not necessitate to define a reference group, generates 95% confidence intervals (95% CI) for each category in the analysis, and expresses hazard ratios (HRs) relative to the average risk in the whole study population. Otherwise, relative risk of death in relation to eGFR, as captured by odds ratios (ORs) or HRs, was expressed per 1-SD decrement in eGFR. Racial differences in NHANES were assessed from the race-by-CKD stage or race-by-eGFR interaction term, as appropriate. The proportional hazard assumption was tested by time-by-variable interaction terms. Finally, in NHANES, the performance of the race-free eGFR equations in their association with mortality was evaluated by the area under curve (AUC).

### Role of the funding source

The funder of the study had no role in the study design, data collection, data analysis, data interpretation, or the writing of the report. All authors collectively had the final responsibility for the decision to submit the manuscript. DWA and JAS vouch for the integrity of the data and the precision of the statistical calculations.

## Results

### Characteristics of participants

#### African-PREDICT and FLEMENGHO

Black compared to White African-PREDICT participants (Table 1 and Supplementary Table 3) had similar sex and age distribution. However, Blacks had lower BMI (24.8 *vs* 25.7 kg/m^2^), but higher systolic/diastolic BPs (121.0/80.2 *vs* 119.0/78.0 mm Hg). Furthermore, Blacks had lower serum creatinine (0.72 *vs* 0.76 mg/dL) and cystatin C (0.66 *vs* 0.69 mg/L) and higher eGFR_cr_ and eGFR_cys_ (*p* ≤ 0.041). mCl_cr_ standardised to BSA was similar in African-PREDICT Black and White participants (133.0 *vs* 134.4 mL/min/1.73 m^2^). White African-PREDICT and White FLEMENGHO participants (Supplementary Table 4) had widely different distributions of age, BMI (Supplementary Figure 1) and systolic, but not diastolic BP (Supplementary Figure 2), or serum creatinine (Supplementary Figure 3). mCl_cr_ standardised to BSA was lower in White Flemish than White South Africans (95.9 *vs* 134.4 mL/min/1.73 m^2^).

**Table 1.** Characteristics of participants by cohort and race.

Characteristics	African-PREDICT		FLEMENGHO	NHANES		Mbuja Mayi
Racial group	Blacks	Whites	Whites	Blacks	Non-Blacks	Blacks
Number in group	341	380	709	1760	7931	401
Women, n (%)	176 (51.6)	189 (49.7)	354 (49.9)	891 (50.6)	3882 (48.9)	129 (32.2)
Median age, years	25.0 (22.5–27.0)	25.0 (22.0–28.0)	49.5 (40.4–59.5)	45 (33–61)	50 (35–67)^§^	54.1 (44.7–63.5)
Serum creatinine, mg/dL	0.72 (0.18)	0.76 (0.22)^†^	0.95 (0.18)	0.97 (0.68)	0.85 (0.38)^§^	1.19 (0.39)
Serum cystatin C, mg/L	0.66 (0.14)	0.69 (0.19)*	…	0.80 (0.50)	0.82 (0.31)*	…
CKD-EPI 2021 derived from						
Creatinine, mL/min/1.73 m2	123.5 (14.7)	119.0 (18.7)^‡^	87.4 (16.9)	92.3 (23.5)	97.5 (22.7)^§^	73.2 (19.3)
CKD stages 1/2/≥3, %	96.2/3.5/0.3	90.0/10.0/0^‡^	43.4/51.8/4.8	58.6/32.4/8.9	67.1/26.1/6.8^§^	22.9/51.6/25.4
Cystatin C, mL/min/1.73 m2	128.8 (18.3)	125.4 (24.4)*	…	108.3 (24.9)	102.0 (25.6)^§^	…
CKD stages 1/2/≥3, %	98.8/1.2	92.9/7.1/0^§^	…	81.3/13.8/5.0	72.4/19.7/7.9^§^	…
Both markers, mL/min/1.73 m2	129.9 (17.6)	125.7 (23.4)^†^	…	104.1 (23.6)	103.7 (23.9)	…
CKD stages 1/2/≥3, %	99.4/0.6	92.6/7.4^§^	…	78.6/16.1/5.3	75.6/18.5/5.9*	…
EKFC 2023 derived from						
Creatinine, mL/min/1.73 m2	109.7 (13.1)	105.6 (16.1)^‡^	80.3 (15.8)	84.5 (21.9)	88.7 (22.0)^§^	68.6 (18.8)
CKD stages 1/2/≥3, %	91.5/8.2/0.3	80.8/19.2/0^§^	27.4/63.6/9.0	44.0/42.2/13.8	51.6/37.2/11.3^§^	18.2/48.4/33.4
Cystatin C, mL/min/1.73 m2	115.7 (10.0)	113.3 (14.7)*	…	100.0 (20.5)	95.1 (21.0)^§^	…
CKD stages 1/2/≥3/, %	99.4/0.6/0	92.9/7.1/0^§^	…	72.7/22.8/4.5	62.8/30.3/6.9^§^	…
Both markers, mL/min/1.73 m2	112.7 (10.4)	109.5 (14.1)^‡^	…	92.2 (20.0)	91.9 (20.6)	…
CKD stages 1/2/3/≥3, %	98.2/1.8/0	89.2/10.8/0^§^	…	62.0/31.0/7.0	58.7/33.3/8.0*	…
Creatinine clearance						
Measured, mL/min	132.9 (77.2)	146.5 (82.4)*	104.9 (36.2)	…	…	…
Standardised, mL/min/1.73 m2	133.0 (75.2)	134.4 (74.3)	95.9 (29.2)	…	…	…

Values are mean (SD), median (IQR) or number of women (%). Chronic kidney disease (CKD) is classified based on eGFR according to the 2024 KDIGO criteria. Creatinine clearance was standardised to body surface area calculated by the Du Bois formula. An ellipsis indicates not measured. Conversion factors: creatinine from mg/dL to µmol/L, multiply by 88.42; cystatin C from mg/L to nmol/L, multiply by 74.9; eGFR and creatinine clearance from mL/min/1.73 m^2^ to mL/s/1.73 m^2^, multiply 0.0167. Significance of the racial difference in African-PREDICT and NHANES: *p ≤ 0.05; ^†^*p* ≤ 0.01; ^‡^*p* ≤ 0.001; ^§^*p* ≤ 0.0001.

#### NHANES participants

The NHANES participants included 1760 Black and 7931 non-Black individuals (Table 1 and Supplementary Table 4), of whom 2656 (33.5%) were Hispanics and 5275 (66.5%) Whites. Compared to non-Blacks Black participants were younger (46.9 *vs* 51.0 years) and had higher BMI (29.6 *vs* 28.1 kg/m^2^), systolic/diastolic BPs (128.7/73.5 *vs* 126.1/71.1 mm Hg) and antihypertensive treatment rates (28.0 *vs* 24.0%). Black individuals had higher serum creatinine (0.97 *vs* 0.85 mg/dL), but lower serum cystatin C (0.80 *vs* 0.82 mg/L). The racial differences in eGFR_cr_, eGFR_cys_ and eGFR_cr-cys_ were concordant with the gradients in the serum biomarkers, irrespective of whether CKD-EPI 2021 or EKFC 2023 equations were applied (Table 1 and Supplementary Table 4). Black compared to non-Black NHANES participants had less favourable baseline characteristics in terms of educational attainment, poverty index, and smoking habits (Supplementary Table 4).

#### Patients hospitalised in Mbuji Maji

Of 401 admitted patients, 89 (22.2%) died in the hospital and 312 (77.8%) were discharged alive (Supplementary Table 5). Systolic/diastolic BPs were severely elevated in all patients, but more in deceased than surviving patients (188.7/110.7 *vs* 175.5/104.4 mm Hg), who also had higher antihypertensive treatment rates (42.6 *vs* 27.0%). The higher serum creatinine in deceased patients was mirrored by lower eGFR_cr_, as derived by the CKD-EPI 2021 (59.4 *vs* 77.2 mL/min/1.73 m^2^) or 2023 EKFC (56.2 *vs* 72.2 mL/min/1.73 m^2^) equations.

### Agreement between eGFR equations

The agreement between eGFRs as derived by various equations was studied by contrasting the CKD-EPI and EKFC formulas, eGFR estimates in Blacks *vs* non-Blacks, and by assessing the impact of the eGFR equations on CKD staging.

#### Comparison of eGFR equations between consortia

All ICCs between eGFRs derived by the EKFC *vs* the CKD-EPI equations were >0.80 (Supplementary Table 6). However, eGFR_cr_, eGFR_cys_ and eGFR_cr-cys_ were consistently greater using the EKFC 2023 compared to the CKD-EPI 2021 equations (Supplementary Table 6). In Black and White African-PREDICT participants, bias (95% LA) ranged from 12.1 (–8.76 to 33.0) mL/min/1.73 m^2^ to 17.2 (1.94 to 32.4) mL/min/1.73 m^2^. In Black and non-Black NHANES and White FLEMENGHO participants, bias ranged from 6.89 (–7.68 to 21.5) mL/min/1.73 m^2^ to 11.9 (–0.18 to 24.0) mL/min/1.73 m^2^.

#### Comparison of eGFR equations between races

ICCs for eGFR between Black and White participants were >0.80 for CKD-EPI and ranged from 0.59 to 0.77 for EKFC equations (Supplementary Table 7). Using the CKD-EPI 2021 equations (Supplementary Table 7) with eGFR_cr-cys_ as reference, no racial differences were detected in African-PREDICT participants with biases (95% LA) ranging from −6.75 (–29.1 to 15.6) to −0.27 (–15.4 to 14.8) mL/min/1.73 m^2^ (*p* ≥ 0.14). In NHANES, for eGFR_cr_, bias was −11.9 (–29.7 to 6.00) mL/min/1.73 m^2^ in Blacks and −6.22 (–24.6 to 12.2) mL/min/1.73 m^2^ in non-Blacks (*p* < 0.0001) and for eGFR_cys_, 4.15 (–13.8 to 22.1) and −1.68 (–17.5 to 14.2) mL/min/1.73 m^2^ (*p* < 0.0001), respectively. The EKFC 2023 equations were applied with eGFR_cys_ as reference and eGFR_cr_ as test (Supplementary Table 7). In African-PREDICT, bias was −6.03 (–27.6 to 14.6) mL/min/1.73 m^2^ in Blacks and −7.68 (–31.6 to 16.2) mL/min/1.73 m^2^ in Whites (*p* = 0.052). In NHANES biases were −15.2 (–42.4 to 11.4) and −6.50 (–31.2 to 18.2) mL/min/1.73 m^2^ in Blacks and non-Blacks (*p* < 0.0001), respectively.

#### Impact of intraindividual variability on CKD classification

Applying the CKD-EPI 2021 equations (Table 2), revealed that fewer than 5% of the young and healthy African-PREDICT participants moved up or down the KDIGO eGFR classification, if eGFR_cr_ or eGFR_cys_ were used for categorisation instead of eGFR_cr-cys_. Only in African-PREDICT participants moving down the racial difference was significant (*p* = 0.040). However, in NHANES (Table 2), if eGFR_cr-cys_ was replaced by eGFR_cr_, 431 Blacks (25.1%) and 1042 non-Blacks (13.1%) participants moved up to a higher (worse) CKD grade and 39 Black (2.2%) and 586 non-Black (7.4%) participants, if eGFR_cys_ was applied (*p* < 0.0001 for racial differences).

**Table 2.** Reclassification of Blacks and non-Blacks according to KDIGO eGFR stage using eGFR_cr-cys_ as reference.

Research consortium Study population Equations compared	Participants moving to higher (worse) stage			Participants moving to lower (better) stage		
	Blacks	Non-Blacks	*p* value	Blacks	Non-Blacks	*p* value
CKD-EPI 2021						
African-PREDICT						
eGFRcr *vs* eGFRcr-cys	12 (3.5)	17 (4.5)	0.57	0 (0.0)	7 (1.8)	0.016
eGFRcr *vs* eGFRcr-cys	4 (1.2)	9 (2.4)	0.27	2 (0.6)	10 (2.6)	0.040
NHANES						
eGFRcr *vs* eGFRcr-cys	431 (24.5)	1042 (13.1)	<0.0001	17 (1.0)	301 (3.8)	<0.001
eGFRcys *vs* eGFRcr-cys	39 (2.2)	586 (7.4)	<0.0001	92 (5.2)	176 (2.2)	<0.001
EKFC 2023						
African-PREDICT						
eGFRcr *vs* eGFRcr-cys	25 (7.3)	37 (9.7)	0.29	1 (0.3)	5 (1.3)	0.22
eGFRcys *vs* eGFRcr-cys	1 (0.3)	6 (1.6)	0.13	5 (1.5)	20 (5.3)	0.007
NHANES						
eGFRcr *vs* eGFRcr-cys	448 (25.5)	985 (12.4)	<0.0001	10 (0.6)	163 (2.1)	<0.0001
eGFRcys *vs* eGFRcr-cys	13 (0.7)	224 (2.8)	<0.0001	243 (13.8)	640 (8.1)	<0.0001

Values are the number of reclassified individuals (%). eGFR_cr_/eGFR_cys_/eGFR_cr-cys_ indicate eGFR derived serum creatinine/cystatin C/both. The number of Blacks and non-Blacks participants amounted to 341 and 380 in African-PREDICT and to 1760 and 7931 in NHANES. eGFR derived from both serum creatinine and serum cystatin C is used as reference. The *p* values refer to the racial differences. The eGFR equations are listed in the Supplementary Tables 1–2 for CKD-EPI 2021 and EKFC 2023.

Applying the EKFC 2023 equations, showed that if eGFR_cr_ was applied instead of eGFR_cr-cys_, 25 Blacks (7.3%) and 37 Whites (9.7%) in African-PREDICT and 448 Blacks (25.5%) and 983 non-Blacks (12.4%) in NHANES moved up to a higher (worse) eGFR stage with a significant racial difference in NHANES (*p* < 0.0001). Applying eGFR_cys_ instead of eGFR_cr-cys_ in African-PREDICT moved 5 (1.5%) Blacks and 20 (5.3%) Whites up to a lower KDIGO stage (*p* = 0.0070). In NHANES, this moved 243 Blacks (13.8%) and 640 non-Blacks (8.1%) to a lower (better) KDIGO stage (*p* < 0.0001).

### mCl_cr_ versus eGFR

**Figure 2.**
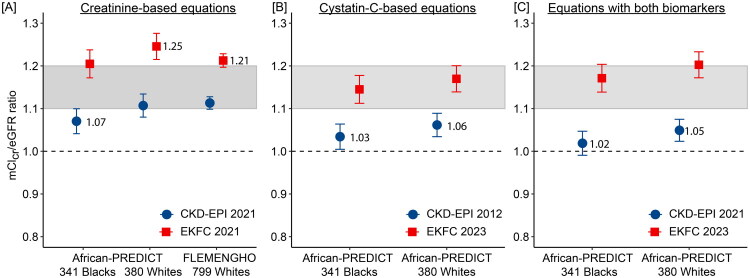
Ratio of measured creatinine clearance to eGFR derived by the CKD-EPI 2021 and EKFC 2023 equations. The equations to compute eGFR are given for CKD-EPI 2021 and EKFC 2023 in the Supplementary Tables 1–2. The mCl_cr_/eGFR ratios for creatinine-based eGFRs are depicted in (a), for cystatin C-based eGFRs in (b), and for equations including both serum biomarkers in (c). The shaded area in the three panels reflects GFR uncorrected for the proximal tubular creatinine secretion, resulting in a mCl_cr_/eGFR ratio ranging from 1.1 to 1.2. mCl_cr_ is standardised to 1.73 m^2^ body surface area. Plotted values are means ± 95% confidence interval. For ratios falling outside the band, the mean is given alongside the plotted value. For the same eGFR equation, none of the racial differences attained statistical significance (*p* ≥ 0.10).

mCl_cr_/eGFR ratios were derived for eGFR_cr_ (Figure 2a), eGFR_cys_ (Figure 2b) and eGFR_cr-cys_ (Figure 2c) in African-PREDICT and FLEMENGHO participants. The shaded area in the three panels reflects eGFR uncorrected for the proximal tubular creatinine secretion, resulting in a mCl_cr_/eGFR ratio ranging from 1.1 to 1.2. Applying the CKD-EPI 2021 equations, the mCl_cr_/eGFR ratio was significantly less than 1.1 in African-PREDICT Blacks for mCl_cr_/eGFR_cr_ (1.07) and in African-PREDICT Black and White participants for mCl_cr_/GFR_cys_ (1.03 and 1.06) and for mCl_cr_/eGFR_cr-cys_ (1.02 and 1.05). Considering the EKFC 2023 equations (Figure 2), the mCl_cr_/eGFR_cr_ ratios were greater than 1.2 in White participants in African-PREDICT (1.25) and FLEMENGHO (1.21). For the same eGFR equation, none of the racial differences attained statistical significance (p ≥ 0.10).

**Figure 3.**
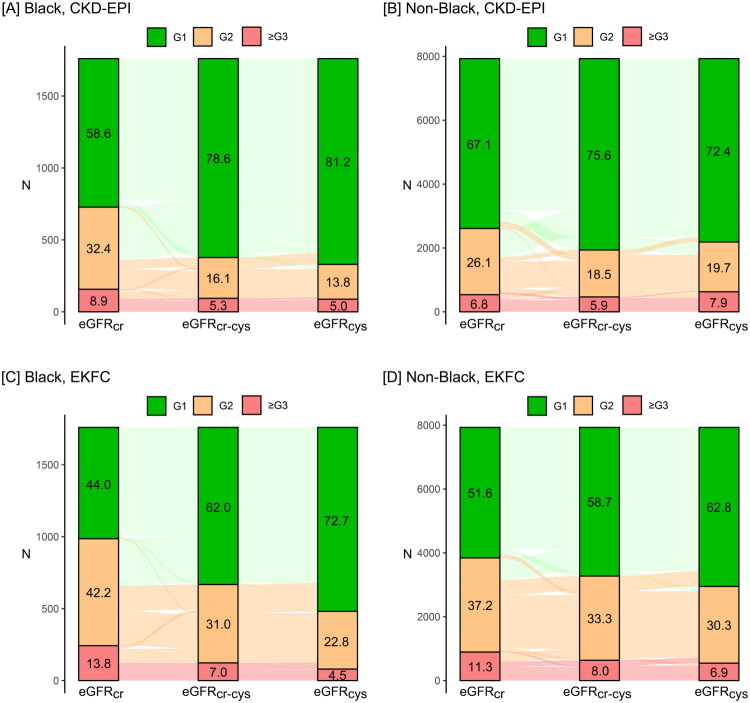
Reclassification of chronic kidney disease grades in NHANES. Numbers in green, orange and red bars are percentages of chronic kidney disease grades (CKD) according to the 2024 KDIGO classification based on eGFR (≥90, 60–89, and <60 mL/min/1.73m^2^) from serum creatinine (eGFR_cr_), serum cystatin C (eGFR_cys)_ or both (eGFR_cr-cys_). Non-Blacks include 2656 Hispanics and 5275 Whites. CKD-EPI denotes Chronic Kidney Disease Epidemiology Collaboration and EKFC the European Kidney Function Consortium. The κ statistic, given with 95% confidence interval, is a measure of the consistency in the distribution of CKD grades between adjacent classifications. κ values of >0.40, >0.60 and >0.80 indicate moderate, substantial and almost perfect agreement.

### Association of mortality with CKD

#### NHANES

Over a median follow-up of 16.8 years (interquartile range,15.0–18.6; 5th–95th percentile interval, 4.3–20.3 years), all-cause, cardiovascular and renal mortality amounted to 455 (25.9%), 143 (8.1%) and 17 (0.97%) deaths among 1760 Blacks and to 2262 (28.5%), 731 (9.2%) and 49 (0.62%) deaths among 7931 non-Blacks.

All Cox models complied with the proportional hazards assumption as assessed by the time-by-variable interaction (0.16≤*p* ≤ 0.90). Table 3 lists the multivariable-adjusted HRs expressing the risk of death or fatal cardiovascular complications by CKD stage compared to the average risk in all NHANES participants. Irrespective of the equation applied for the derivation of eGFR and subsesquently the CKD grade, all HRs were significant, lower than unity for CKD grade 1 and greater than unity for CKD grade ≥3 with a significant trend *p* value from stage 1 to ≥3 (*p* < 0.0001) and without any racial difference (0.11≤*p* ≤ 0.98). These findings were consistent with the HR expressing the risk of total, cardiovascular or renal mortality per 1-SD decrement in eGFR_cr_ (Supplementary Table 8), eGFR_cys_ (Supplementary Table 9) or eGFR_cr-cys_ (Supplementary Table 10). Subgroup analyses of total and cardiovascular mortality categorised by sex, age (<60 *vs* ≥ 60 years) or the presence of diabetes (Supplementary Figures 4–5) were confirmatory, but the relative risk conferred by eGFR was greater in non-diabetic compared to diabetic patients. Considering total and cardiovascular mortality (Supplementary Table 11), eGFR_cr_ did not consistently increase the AUC in Black and non-Black participants on top of the base model including as covariables sex, age, BMI and MAP, smoking, educational attainment and the poverty index. In both racial groups, eGFR_cys_ and eGFR_cr-cys_ enlarged the AUC for all-cause and cardiovascular mortality (*p* ≤ 0.050), albeit with a small amount, ranging from 0.009 to 0.023 in Blacks and from 0.004 to 0.007 in non-Blacks (Supplementary Table 11).

**Table 3.** Mortality in NHANES by CKD stage and race

Biomarkers Derivation of eGFR Consortium CKD grade	N	Total mortality		Cardiovascular mortality	
		N endpoint	HR (95% CI)	N endpoint	HR (95% CI)
**Serum creatinine**					
CKD-EPI		*p*-ir = 0.15		*p*-ir = 0.16	
G1	6353	1042	0.82 (0.77–0.88)	298	0.78 (0.70–0.88)
G2	2644	1115	0.90 (0.85–0.95)	367	0.89 (0.81–0.97)
≥G3	694	560	1.35 (1.26–1.44)	209	1.44 (1.29–1.62)
EKFC		*p*-ir = 0.11		*p*-ir = 0.43	
G1	4863	457	0.90 (0.82–0.99)	126	0.96 (0.81–1.14)
G2	3691	1372	0.86 (0.82–0.91)	425	0.80 (0.73–0.88)
≥G3	1137	888	1.28 (1.19–1.38)	323	1.29 (1.13–1.48)
**Serum cystatin C**					
CKD-EPI		*p*-ir = 0.33		*p*-ir = 0.36	
G1	7174	1009	0.65 (0.60–0.69)	283	0.62 (0.55–0.70)
G2	1801	1071	0.93 (0.88–0.98)	356	0.91 (0.83–1.00)
≥G3	716	637	1.67 (1.57–1.79)	235	1.77 (1.58–1.98)
EKFC		*p*-ir = 0.98		*p*-ir = 0.83	
G1	6260	522	0.59 (0.53–0.65)	131	0.59 (0.49–0.71)
G2	2805	1627	0.92 (0.87–0.97)	532	0.89 (0.80–0.98)
≥G3	626	568	1.85 (1.71–2.01)	211	1.92 (1.66–2.21)
**Creatinine + cystatin C**					
CKD-EPI		*p*-ir = 0.80		*p*-ir = 0.40	
G1	7376	1148	0.67 (0.63–0.72)	321	0.62 (0.55–0.69)
G2	1754	1067	0.93 (0.88–0.99)	360	0.93 (0.85–1.02)
≥G3	561	502	1.59 (1.48–1.71)	193	1.74 (1.54–1.95)
EKFC		*p*-ir = 0.12		*p*-ir = 0.58	
G1	5748	465	0.70 (0.64–0.78)	122	0.74 (0.62–0.90)
G2	3183	1570	0.87 (0.82–0.92)	497	0.82 (0.74–0.90)
≥G3	760	682	1.63 (1.50–1.77)	255	1.65 (1.43–1.91)

*p*-ir denotes the significance of the racial difference (Blacks *vs* non-Blacks). Chronic kidney disease is staged based on eGFR according to the 2024 KDIGO Guidelines (≥90, 60–89, and <60 mL/min/1.73m^2^). CKD stages are modelled by the deviation-from-mean coding, so that the hazard ratios (HRs) expressed risk relative to the average risk in the whole NHANES cohort. HRs, given with 95% confidence interval, are adjusted for sex, age, BMI, MAP, smoking, educational attainment and the poverty index. All *p* values for trend from G1 to ≥ G3 are <0.0001.

#### Mbuji Mayi, DMC

In-hospital mortality in Mbuji Mayi was significantly related to eGFR_cr_ as determined by the CKD-EPI 2021 and EKFC 2023 equations. In unadjusted analyses, the ORs associated with eGFR_cr_ were 2.38 (95% CI, 1.81–3.57) and 2.30 (1.77–2.98). With adjustments applied for sex, age, BMI, and antihypertensive treatment status, the corresponding ORs were 2.54 (1.81–3.57) and 2.69 (1.88–3.86), respectively.

## Discussion

eGFR is the tool par excellence for the management of CKD in patients with established disease [31]. The CKD-EPI [8] and EKFC [11] consortia recommend the use of race-free eGFR equations. The current study evaluated race-free eGFR equations in the detection of the early stages of CKD from the perspective of the prevention of progression to established CKD. The main study outcomes obtained in Black and non-Black individuals enrolled in racially and regionally widely diverse community-based studies, can be summarised along five lines. First, as evidenced by the ICCs and κ statistics, there is substantial agreement between the various race-free eGFR formulations in Blacks and non-Blacks. Second, the Bland-Altman analysis [29], highlighted large bias and wide limits of agreement, signifying substantial intra-individual variability, in eGFR estimates, which in NHANES was greater in Blacks than non-Blacks. Third and most relevant for the clinical application of eGFR in the early prevention of CKD, is that the considerable intra-individual variability in eGFR estimates led to reclassification of individuals. Applying the recommended eGFR_cr-cys_ [8,11] to adults from 18 to 80 years old, revealed that up to 25% of Blacks and up to 13% non-Blacks moved up to a higher (worse) CKD grade, whereas up to 14% of Blacks and up to 8% of non-Blacks moved down to a lower (better) CKD stage. In both scenarios, Blacks fared worse than Whites. Fourth, the race-free eGFR estimates in half of the tests (7/14) over- or under-estimated the glomerular filtration rate (GFR) uncorrected for the proximal tubular creatinine secretion. Finally, despite the large intra-individual variability between eGFR formulations and the ensuing CKD grades, both CKD stage and eGFR, irrespective of their derivation, predicted total and cardiovascular mortality in NHANES and the in-hospital mortality in the Mbuji Mayi cohort, albeit with little model refinement on top of other risk factors, as shown by the AUC in NHANES.

According to the CKD-EPI Collaboration [7–9], the race-free eGFR equations that incorporate creatinine and cystatin C are more accurate and lead to smaller racial differences compared to race-free eGFR estimates based only on either serum creatinine or cystatin C. The EKFC eGFR_cys_ equation [11] has the same mathematical form as the EKFC eGFR_cr_ formula [10], but has a scaling factor for cystatin C that does not differ according to race or sex. In cohorts from Europe, the United States and Africa, this equation improved the accuracy of the assessment of measured GFR over that of commonly used equations [10,11]. Because of availability of the technology in laboratories worldwide, routine measurement of serum creatinine remains the cornerstone in the derivation of eGFR. The race-free equations do amend the overestimation of eGFR_cr_ in Blacks, but compared to eGFR_cr-cys_ and eGFR_cys_ generated lower estimates of eGFR and therefore higher (worse) CKD grades. In relation to total and cardiovascular mortality, eGFR_cr_ did not consistently increase the AUC in Black and non-Black participants on top of the base model. Furthermore, by using eGFR_cr-cys_ (CKD-EPI) or eGFR_cys_ (EKFC) as reference, the underestimation of eGFR in NHANES was greater in Black than White individuals, so that apparently the racial differences are not completely levelled off. The common assumption is that muscularity and genetic ancestry underly the higher serum creatinine levels in Blacks compared to non-Blacks [5,6]. However, in a sample of 1248 CKD patients representative of American patients [32], the use of serum creatinine to determine eGFR without considering race or genetic ancestry introduced systematic misclassification that could not be eliminated even when numerous external determinants of serum creatinine were accounted for. The lower eGFR value of either eGFR_cr_ or eGFR_cys_ provided the most accurate and less biased estimate compared to the measured GFR [33].

While current recommendations favour the applications of eGFR_cys_ and eGFR_cr-cys_ over eGFRcr [7–11], some constraints have to be kept in mind [34]. In a study of 8058 inhabitants of Groningen (The Netherlands) [35], older age, male sex, greater weight and height, current cigarette smoking, and higher serum C-reactive protein levels were independently associated with higher serum cystatin C. Finally, serum cystatin C measurements, while technically feasible, are often not on the test repertoire of laboratories, may not be covered by health insurance, or are too expensive for routine application compared to serum creatinine [36,37]. The cost-effectiveness of measuring routinely eGFR_cys_ should therefore be further evaluated, especially in populations with limited healthcare coverage.

### Strengths and limitations

The present study evaluated various eGFR formulations with CKD prevention as perspective, whereas most previous studies addressed the application of eGFR as a tool in the management of established CKD. The multi-ethnic and regional diversity of the studies currently analysed provided a database appropriate to assess race-free eGFR equations, thereby enhancing generalisability. Nevertheless, the present results must be interpreted within the context of several limitations. First, although measurement of serum creatinine using isotope-dilution mass spectrometry for calibration [24] was used in all studies, inevitably there must have been undocumented differences in the serum creatinine measurement not only between studies, but also over time within studies. However, with the exception of the evaluation of the mCl_cr_/eGFR ratio, all analyses were stratified by cohort. Second, the race-free eGFR equations were tested against mCl_cr_. Given the race-specific differences in tubular creatinine handling, this approach is inferior to the use of other markers used to assess measured GFR, such as iohexol, inulin, pentaacetic acid or radio-active labelled molecules. Third, the NHANES results on renal mortality as presented in the Supplementary Tables 8–10 should be viewed as exploratory, given the low death rate due to renal disease. Underreporting of renal mortality might be an issue in relating baseline NHANES data with the cause of death as recorded in the National Death Index. Fourth, CKD grading in the current study did not involve assessment of albuminuria. It did also not differentiate between the complementary high-risk and population-based approaches in screening for early-stage CKD grades [15]. However, serum creatinine and eGFR are commonly reported among the biochemical analytics, which are part of any health check-up in primary and specialist care in most developed countries, making eGFR de facto a tool for opportunistic CKD screening with possibly greater health gains than the high-risk approach [38]. Finally, whether or not the association of mortality with eGFR as derived from the new race-free equations might be closer than the race-inclusive equations was beyond the scope of this article. However, in the Mbuji Mayi cohort, the adjusted OR expressing the risk of in-hospital mortality per 1-SD decrement in eGFR_cr_ derived by Modification of Diet in Renal Disease Study Group equation [3] was 1.96 (95% CI 1.48–2.66) [21], to be compared with 2.54 (1.81–3.57) and 2.69 (1.88–3.86) according to the race-free CKD-EPI and EKFC equations.

## Conclusions

The current study moves the field forward by application of the race-free eGFR formulations to individuals recruited from communities in South Africa, Belgium, the Democratic Republic of Congo and the United States. While mortality outcomes do support use of race-free eGFR equations in population-based studies, large intraindividual variability between eGFR estimates might lead to KDIGO^2^ eGFR stage misclassification and calls for caution in opportunistic or systematic screening in asymptomatic individuals with prevention as objective. Taken together, all available evidence highlights the need of further research to optimise the application of eGFR for screening and prevention of CKD at the population level. One suggestion that perhaps deserves to be evaluated is whether race-stratified eGFR_cr_ equations, i.e. separate equations for Blacks and non-Blacks might provide the least biased and most accurate eGFRcr estimates [37].

## Supplementary Material

Supplemental Material

## Data Availability

The summary statistics reported in this article are publicly available. The NHANES datasets are freely available at https://www.cdc.gov/nchs/nhanes/index.htm. Informed consent given by study participants enrolled in African-PREDICT, FLEMENGHO or in the Mbuji Mayi cohort did not include data sharing with third parties. However, anonymised data can be made available to investigators for targeted non-commercial research based on a motivated request to be submitted to JA Staessen and pending ethical clearance by the Principal Investigators and the Institutional Ethics Review Boards, involved in the current study.
