# Supplementary material for: Evaluation of race-free eGFR equations in individuals of different ethnicity
Source: Blood Press. 2025 Jul 15;34(1):2533456. doi: 10.1080/08037051.2025.2533456 (PMC12315838; doi:10.1080/08037051.2025.2533456)
Supplement: Supplemental Material [file IBLO_A_2533456_SM3528.doc]

**Blood Pressure**

**Supplementary material**

Supplement to: *Evaluation of race-free eGFR equations in individuals of different ethnicity*

DW An, GG Mokwatsi, DY Zhang, DS Martens, YL Yu, BS Chori, AN Odili, R Kruger, LF Gafane-Matemane,
J Siwy, A Latosinska, H Mischak, CMC Mels, AE Schutte, JR M’Buyamba-Kabangu, TS Nawrot, Y Li,
JA Staessen

**Table of contents**

**page**

***Table 1:*** Race-free eGFR equations proposed by the Chronic Kidney Disease Epidemiology Collaboration p2

***Table 2:*** Race-free eGFR equations proposed by the European Kidney Function Consortium p3

***Table 3:*** Characteristics of African-PREDICT and FLEMENGHO participants p4

***Table 4:*** Baseline characteristics (1999-2004) of NHANES participants by race p5

***Table 5:*** Characteristics of patients on hospital admission in Mbuji Mayi (2001-2003) p6

***Table 6:*** Agreement between the CKD-EPI 2021 and EKFC 2023 race-free equations p7

***Table 7:*** Comparison of race-free eGFR equations between Black and non-Black individuals p8

***Table 8:*** Association of mortality with eGFR derived from serum creatinine in NHANES p9

***Table 9:*** Association of mortality with eGFR derived from serum cystatin C in NHANES p10

***Table 10:*** Association of mortality with eGFR derived from serum creatinine and cystatin C in NHANES p11

***Table 11:*** AUC for mortality in relation to race-free eGFR equations in NHAHES p12

***Figure 1:*** Distributions of age and body mass index in African-PREDICT and FLEMENGHO participants p13

***Figure 2:*** Distributions of systolic and diastolic blood pressure in Black and White African-PREDICT
and White FLEMENGHO participants p14

***Figure 3:*** Distributions of serum creatinine and serum cystatin C in Black
and White African-PREDICT and White FLEMENGHO participants p15

***Figure 4:*** Subgroup analysis of the association of all-cause mortality with eGFR derived from
both serum creatinine and cystatin C in NHANES p16

***Figure 5:*** Subgroup analysis of the association of cardiovascular mortality with eGFR derived from
both serum creatinine and cystatin C in NHANES p17

***Table 1:***

**Race-free eGFR equations proposed by the Chronic Kidney Disease Epidemiology Collaboration**

| **Serum biomarker** | **Sex** | **Creatinine (mg/dL)** | **Cystatin-C (mg/L)** | **Equation for estimating eGFR** |
| --- | --- | --- | --- | --- |
| **Creatinine** | female | ≤0.7 | … | 142 × (cr / 0.7)-0.241 × 0.9938age × 1.012 |
|  |  | >0.7 | … | 142 × (cr / 0.7)-1.200 × 0.9938age × 1.012 |
|  | male | ≤0.9 | … | 142 × (cr / 0.7)-0.302 × 0.9938age |
|  |  | >0.9 | … | 142 × (cr / 0.7)-1.200 × 0.9938age |
| **Cystatin C** | female | … | ≤0.80 | 133 × (cys / 0.8)-0.499 × 0.996age × 0.932 |
|  |  | … | >0.80 | 133 × (cys / 0.8)-1.328 × 0.996age × 0.932 |
|  | male | … | ≤0.80 | 133 × (cys / 0.8)-0.499 × 0.996age |
|  |  | … | >0.80 | 133 × (cys/ 0.8)-1.328 × 0.996age |
| **Both biomarkers** | female | ≤0.7 | ≤0.80 | 130 × (cr / 0.7)-0.219 × (cys / 0.80)-0.323 × 0.9961age |
|  |  | ≤0.7 | >0.80 | 130 × (cr / 0.7)-0.219 × (cys / 0.80)-0.778 × 0.9961age |
|  |  | >0.70 | ≤0.80 | 130 × (cr / 0.7)-0.554 × (cys / 0.80)-0.323 × 0.9961age |
|  |  | >0.70 | >0.80 | 130 × (cr / 0.7)-0.544 × (cys / 0.80)-0.778 × 0.9961age |
|  | male | ≤0.90 | ≤0.80 | 130 × (cr / 0.7)-0.144 × (cys / 0.80)-0.323 × 0.9961age |
|  |  | ≤0.90 | >0.80 | 130 × (cr / 0.7)-0.144 × (cys / 0.80)-0.778 × 0.9961age |
|  |  | >0.90 | ≤0.80 | 130 × (cr / 0.7)-0.554 × (cys / 0.80)-0.323 × 0.9961age |
|  |  | >0.90 | >0.80 | 130 × (cr / 0.7)-0.554 × (cys / 0.80)-0.778 × 0.9961age |

The CKD-EPI 2021 equations are applicable to adults (≥18 y), in which *cr* and *cys* indicate serum creatinine (mg/dL) and serum cystatin C (mg/L). For more information, see Inker LA et al. *N Engl J Med*. 2021;385:1737-49.

***Table 2:***

**Race-free eGFR equations proposed by the European Kidney Function Consortium**

| **Serum biomarker** | **Age (y)** | **Sex** | **Condition** | **Equation for estimating eGFR** |
| --- | --- | --- | --- | --- |
| **Creatinine** | 18-40 | female | cr / Q < 1.0 | 107.3 × (cr / Q) )-0.322 |
|  |  |  | cr / Q ≥ 1.0 | 107.3 × (cr / Q) )-1.132 |
|  |  | male | cr / Q < 1.0 | 107.3 × (cr / Q) )-0.322 |
|  |  |  | cr / Q ≥ 1.0 | 107.3 × (cr / Q) )-1.132 |
|  | >40 | female | cr / Q < 1.0 | 107.3 × (cr / Q) )-0.322 × 0.990(age – 40) |
|  |  |  | cr / Q ≥ 1.0 | 107.3 × (cr / Q) )-1.132 × 0.990(age – 40) |
|  |  | male | cr / Q < 1.0 | 107.3 × (cr / Q) )-0.322 × 0.990(age – 40) |
|  |  |  | cr / Q ≥ 1.0 | 107.3 × (cr / Q) )-1.132 × 0.990(age – 40) |
| **Cystatin C** | 18-40 | na | cys / 0.83 < 1.0 | 107.3 × (cyst / 0.83)-0.322 |
|  |  |  | cys / 0.83 ≥ 1.0 | 107.3 × (cys / 0.83)-1.132 |
|  | >40 | na | cys / 0.83 < 1.0 | 107.3 × (cys / 0.83)-0.322 × 0.990(age – 40) |
|  |  |  | cys / 0.83 ≥ 1.0 | 107.3 × (cys / 0.83)-1.132 × 0.990(age – 40) |
|  | >50 | na | cys / Q < 1.0 (see footnote) | 107.3 × (cys / Q)-0.322 × 0.990(age – 40) |
|  |  |  | cys / Q ≥ 1.0 (see footnote) | 107.3 × (cys / Q)^-^1.132 × 0.990(age – 40) |
| **Both biomarkers** | … | … | … | arithmetic average of eGFR based on *cr* and *cys* |

*cr* and *cys* indicates serum creatinine (mg/dL) and serum cystatin C (mg/L). Q is population specific for creatinine, but not for cystatin C. In the current study, Q is set at 0.70 mg/dL for women and 0.90 mg/dL for men. For eGFR derived from serum cystatin C in individuals older than 50 years, Q = 0.83 + 0.005 × (age – 50). For more information, see Pottel et al. *N Engl J Med.* 2023; 388: 333-43.

*Table 3:*

**Characteristics of African-PREDICT and FLEMENGHO participants**

| **Characteristic** | **African-PREDICT** | | |  | **FLEMENGHO** | **pAP-FW** |
| --- | --- | --- | --- | --- | --- | --- |
|  | **Blacks** | **Whites** | **pB-W** |  |  |  |
| Number in group | 341 | 380 |  |  | 709 |  |
| Women, n (%) | 176 (51.6) | 189 (49.7) | 0.67 |  | 354 (49.9) | 0.83 |
| Age, y | 25.1 (3.1) | 24.8 (3.0) | 0.33 |  | 49.5 (15.4) | <0.001 |
| Body mass index, kg/m^2^ | 24.8 (5.6) | 25.7 (5.4) | 0.024 |  | 26.4 (4.3) | <0.001 |
| Systolic blood pressure, mm Hg | 121.0 (12.9) | 119.0 (12.5) | 0.034 |  | 128.83 (17.4) | <0.001 |
| Diastolic blood pressure, mm Hg | 80.2 (9.5) | 78.0 (7.8) | <0.001 |  | 79.9 (9.7) | 0.084 |
| Mean arterial pressure, mm Hg | 96.6 (9.9) | 94.4 (8.8) | 0.002 |  | 99.5 (11.3) | <0.001 |
| Current smoking | 79 (23.2) | 82 (21.6) | 0.67 |  | 138 (19.5) | <0.001 |
| Serum creatinine, mg/dL | 0.72 (0.18) | 0.76 (0.22) | 0.005 |  | 0.95 (0.18) | <0.001 |
| Serum cystatin C, mg/L | 0.66 (0.14) | 0.69 (0.19) | 0.027 |  | … | … |
| CKD-EPI 2021 derived from |  |  |  |  |  |  |
| Serum creatinine, mL/min/1.73 m^2^ | 123.5 (14.7) | 119.0 (18.7) | <0.001 |  | 87.4 (16.9) | <0.001 |
| Serum cystatin C, mL/min/1.73 m^2^ | 128.8 (18.3) | 125.4 (24.4) | 0.041 |  | … | … |
| Both serum markers, mL/min/1.73 m^2^ | 129.9 (17.6) | 125.7 (23.4) | 0.008 |  | … | … |
| EKFC 2023 derived from |  |  |  |  |  |  |
| Serum creatinine, mL/min/1.73 m^2^ | 109.7 (13.1) | 105.6 (16.1) | <0.001 |  | 80.3 (15.8) | <0.001 |
| Serum cystatin C, mL/min/1.73 m^2^ | 115.7 (10.0) | 113.3 (14.7) | 0.012 |  | … | … |
| Both serum markers, mL/min/1.73 m^2^ | 112.7 (10.4) | 109.5 (14.1) | <0.001 |  | … | … |
| Creatinine clearance |  |  |  |  |  |  |
| Measured, mL/min | 132.9 (77.2) | 146.5 (82.4) | 0.023 |  | 104.9 (36.2) | <0.001 |
| Standardised, 1.73 m^2^ mL/min | 133.0 (75.2) | 134.4 (74.3) | 0.80 |  | 95.9 (29.2) | <0.001 |

Values are mean (SD) or number of participants with characteristic (%).pB-W refers to the difference between black and white African-PREDICT participants and pAP-FW to the difference between white African-PREDICT and white FLEMENGHO participants. Creatinine clearance was standardised to body surface area calculated by the Du Bois formula. An ellipsis indicates not measured. Mean arterial pressure is diastolic blood pressure + 0.40 × (systolic – diastolic blood pressure). Conversion factors: creatinine from mg/dL to μmol/L, multiply by 88.42; cystatin C from mg/L to nmol/L, multiply by 74.9; eGFR and creatinine clearance from ml/min/1.73 m2 to ml/s/1.73 m2, multiply 0.0167.

*Table 4:*

**Baseline characteristics (1999-2004) of NHANES participants by race**

| **Characteristic** | **Blacks** | **Non-Blacks** | **p value** |
| --- | --- | --- | --- |
| Number in group | 1760 | 7931 |  |
| Women, n (%) | 891 (50.6) | 3882 (48.9) | 0.21 |
| Age, y | 46.9 (17.3) | 51.0 (18.7) | <0.0001 |
| Body mass index, kg/m^2^ | 29.6 (6.98) | 28.1 (5.79) | <0.0001 |
| Systolic blood pressure, mm Hg | 128.7 (21.4) | 126.1 (20.6) | <0.0001 |
| Diastolic blood pressure, mm Hg | 73.5 (13.4) | 71.1 (11.8) | <0.0001 |
| Mean arterial pressure, mm Hg | 95.6 (14.1) | 93.1 (12.3) | <0.0001 |
| Hypertension, n (%) | 885 (50.3) | 3458 (43.6) | <0.0001 |
| Treated hypertension, n (%) | 492 (28.0) | 1901 (24.0) | 0.0006 |
| Diabetes, n (%) | 272 (15.5) | 960 (12.1) | 0.0002 |
| Serum creatinine, mg/dL | 0.97 (0.68) | 0.85 (0.38) | <0.0001 |
| Serum cystatin C, mg/L | 0.80 (0.50) | 0.82 (0.31) | 0.023 |
| CKD-EPI 2021 eGFR derived from |  |  |  |
| Serum creatinine, mL/min/1.73 m^2^ | 92.3 (23.5) | 97.5 (22.7) | <0.0001 |
| Serum cystatin C, mL/min/1.73 m^2^ | 108.3 (24.9) | 102.0 (25.6) | <0.0001 |
| Both serum markers, mL/min/1.73 m^2^ | 104.1 (23.6) | 103.7 (23.9) | 0.50 |
| EKFC 2023 eGFR derived from |  |  |  |
| Serum creatinine, mL/min/1.73 m^2^ | 84.5 (21.9) | 88.7 (22.0) | <0.0001 |
| Serum cystatin C, mL/min/1.73 m^2^ | 100.0 (20.5) | 95.1 (21.0) | <0.0001 |
| Both serum markers, mL/min/1.73 m^2^ | 92.2 (20.0) | 91.9 (20.6) | 0.53 |
| Educational attainment |  |  |  |
| Less than high school, n (%) | 1025 (58.2) | 4346 (54.8) | <0.0001 |
| High school, n (%) | 504 (28.6) | 2007 (25.3) |
| College or equivalent, n (%) | 231 (13.1) | 1578 (19.9) |
| Poverty index |  |  |  |
| ≤1.3, n (%) | 567 (32.2) | 2059 (26.0) | <0.0001 |
| 1.3-3.5, n (%) | 740 (42.0) | 3067 (38.7) |
| >3.5, n (%) | 453 (25.7) | 2805 (35.4) |
| Smoking status |  |  |  |
| Never, n (%) | 871 (49.5) | 3769 (47.5) | <0.0001 |
| Past, n (%) | 285 (16.2) | 2124 (26.8) |
| Current, n (%) | 604 (34.3) | 2038 (25.7) |

Values are mean (SD) or number of participants with characteristic (%). Mean arterial pressure is diastolic blood pressure + 0.40 × (systolic – diastolic blood pressure). Hypertension is a blood pressure of ≥140 mm Hg systolic or ≥90 mm Hg diastolic or the use of antihypertensive drugs. Diabetes is a self-reported diagnosis at the baseline home visit. The poverty index is the ratio of family income to poverty as defined in each survey year by the Department of Health and Human Services, higher values indicating greater affluence. The p value refers to the racial differences. Conversion factors: creatinine from mg/dL to μmol/L, multiply by 88.42; cystatin C from mg/L to nmol/L, multiply by 74.9; eGFR from mL/min/1.73 m2 to mL/s/1.73 m2, multiply 0.0167.

*Table 5:*

**Characteristics of patients on hospital admission in Mbuji Mayi (2001-2003)**

| **Characteristic** | **Deceased** | **Survived** | **p value** |
| --- | --- | --- | --- |
| Number in group | 89 | 312 |  |
| Women, n (%) | 31 (34.8) | 98 (31.4) | 0.54 |
| Age, y | 53.3 (9.9) | 54.3 (9.2) | 0.38 |
| Body mass index, kg/m^2^ | 25.3 (3.2) | 23.8 (2.0) | <0.0001 |
| Systolic blood pressure, mm Hg | 188.7 (16.3) | 175.5 (15.5) | <0.0001 |
| Diastolic blood pressure, mm Hg | 110.7 (8.1) | 104.4 (10.0) | <0.0001 |
| Mean arterial pressure, mm Hg | 141.9 (10.0) | 132.8 (11.4) | <0.0001 |
| Treated for hypertension, n (%) | 24 (27.0) | 133 (42.6) | 0.0076 |
| Diabetes, n (%) | 36 (40.4) | 49 (15.7) | <0.0001 |
| Current smoking, n (%) | 16 (18.0) | 47 (15.1) | 0.50 |
| Serum creatinine, mg/dL | 1.55 (0.69) | 1.09 (0.25) | <0.0001 |
| eGFR derived from serum creatinine |  |  |  |
| CKD-EPI 2021, mL/min/1.73 m^2^ | 59.4 (26.2) | 77.2 (16.8) | <0.0001 |
| EKFC 2023, mL/min/1.73 m^2^ | 56.2 (24.4) | 72.2 (16.9) | <0.0001 |

Values are mean (SD) or number of participants with characteristic (%). Mean arterial pressure is diastolic blood pressure + 0.40 × (systolic – diastolic blood pressure). All patients had hypertension (blood pressure of ≥140 mm Hg systolic or ≥90 mm Hg diastolic or use of antihypertensive drugs). Diabetes is a diagnosis on hospital admission. The p value refers to the difference between deceased and surviving patients. Conversion factors: creatinine from mg/dL to μmol/L, multiply by 88.42; eGFR from mL/min/1.73 m2 to mL/s/1.73 m2, multiply 0.0167.

*Table 6:*

**Agreement between the CKD-EPI 2021 and EKFC 2023 race-free equations**

| **Race   Study group   eGFR equation** |  | **Intraclass correlation coefficient (95% CI)** |  | **Δ (95% LA)**  **CKD-EPI minus EKFC (ml/min1**.**73 m2)** |
| --- | --- | --- | --- | --- |
| **Black individuals** |  |  |  |  |
| African-PREDICT |  |  |  |  |
| eGFRcr |  | 0.97 (0.96–0.98) |  | 13.8 (7.24–20.4) |
| eGFRcys |  | 0.81 (0.77–0.84) |  | 13.1 (-4.94–31.1) |
| eGFRcr-cys |  | 0.86 (0.82–0.88) |  | 17.2 (1.94–32.4) |
| NHANES |  |  |  |  |
| eGFRcr |  | 0.98 (0.98–0.98) |  | 7.80 (-0.87–16.5) |
| eGFRcys |  | 0.95 (0.94–0.95) |  | 8.30 (-6.20–22.8) |
| eGFRcr-cys |  | 0.96 (0.96–0.96) |  | 11.9 (-0.18–24.0) |
| Mbuji Mayi |  |  |  |  |
| eGFRcr |  | 0.99 (0.99–0.99) |  | 4.55 (0.32–8.78) |
| **Non-Black individuals** |  |  |  |  |
| African-PREDICT |  |  |  |  |
| eGFRcr |  | 0.98 (0.97–0.98) |  | 13.3 (5.94–20.7) |
| eGFRcys |  | 0.86 (0.83–0.88) |  | 12.1 (-8.76–33.0) |
| eGFRcr-cys |  | 0.87 (0.84–0.89) |  | 16.2 (-2.99–35.5) |
| FLEMENGHO |  |  |  |  |
| eGFRcr |  | 0.97 (0.96–0.97) |  | 7.12 (-0.78–15.0) |
| NHANES |  |  |  |  |
| eGFRcr |  | 0.98 (0.98–0.98) |  | 8.84 (0.70–17.0) |
| eGFRcys |  | 0.95 (0.95–0.95) |  | 6.89 (-7.68–21.5) |
| eGFRcr-cys |  | 0.96 (0.96–0.96) |  | 11.8 (-0.53–24.2) |

eGFRcr/eGFRcys/eGFRcr-cys indicate eGFR derived from serum creatinine/cystatin C or both biomarkers. Intraclass correlation coefficients (ICC) of >0.80 indicate perfect agreement. Bias (Δ) is computed as eGFR CKD-EPI minus eGFR EKFC. The 95% limits of agreement (95% LA) are Δ ± (1.96 × SD). The eGFR equations are listed in table 1 (p 2) for CKD-EPI 2021 and in table 2 (p 3) for EKFC 2023.

*Table 7:*

**Comparison of race-free eGFR equations between Black and non-Black individuals**

| **Research consortium   Study population   Equations compared** |  | **Intraclass correlation coefficient (95% CI )** | | |  | **Δ (95% LA), ml/min1.73 m2** | | |
| --- | --- | --- | --- | --- | --- | --- | --- | --- |
|  |  | **Blacks** | **Non-Blacks** | **p value** |  | **Blacks** | **Non-Blacks** | **p value** |
| **CKD-EPI 2021** |  |  |  |  |  |  |  |  |
| African-PREDICT |  |  |  |  |  |  |  |  |
| eGFRcr-cys (R) *vs* eGFRcr (T) |  | 0.83 (0.80–0.86) | 0.85 (0.83-0.88) | 0.31 |  | -6.36 (-24.7–12.0) | -6.75 (-29.1–15.6) | 0.61 |
| eGFRcr-cys (R) *vs* eGFRcys (T) |  | 0.92 (0.90–0.94) | 0.95 (0.94-0.96) | 0.0020 |  | -1.10 (-15.0–12.8) | -0.27 (-15.4–14.8) | 0.14 |
| NHANES |  |  |  |  |  |  |  |  |
| eGFRcr-cys (R) *vs* eGFRcr (T) |  | 0.93 (0.92–0.93) | 0.92 (0.92-0.92) | 0.28 |  | -11.9 (-29.7–6.00) | -6.22 (-24.6–12.2) | <0.0001 |
| eGFRcr-cys (R) *vs* eGFRcys (T) |  | 0.93 (0.92–0.93) | 0.95 (0.94-0.95) | <0.0001 |  | 4.15 (-13.8–22.1) | -1.68 (-17.5–14.2) | <0.0001 |
| **EKFC 2023** |  |  |  |  |  |  |  |  |
| African-PREDICT |  |  |  |  |  |  |  |  |
| eGFRcys (R) *vs* eGFRcr (T) |  | 0.59 (0.52–0.66) | 0.69 (0.63-0.74) | 0.010 |  | -6.03 (-26.7–14.6) | -7.68 (-31.6–16.2) | 0.052 |
| NHANES |  |  |  |  |  |  |  |  |
| eGFRcys (R) *vs* eGFRcr (T) |  | 0.79 (0.77–0.81) | 0.83 (0.82-0.83) | <0.0001 |  | -15.5 (-42.4–11.4) | -6.50 (-31.2–18.2) | <0.0001 |

eGFRcr/eGFRcys/eGFRcr-cys indicate eGFR derived from serum creatinine/cystatin C or both biomarkers. Intraclass correlation coefficients (ICC) of >0.80 indicate perfect agreement. Bias (Δ) is computed as eGFR test (T) minus eGFR reference (R). The 95% limits of agreement (95% LA) are Δ ± (1.96 × SD). According to EKFC 2023, eGFRcr-cys is the average of eGFRcr and eGFRcys and eGFRcr-cys is therefore not analysed. The p values refer to the racial differences. The eGFR equations are listed in table 1 (p 2) for CKD-EPI 2021 and in table 2 (p 3) for EKFC 2023.

*Table 8:*

**Association of mortality with eGFR derived from serum creatinine in NHANES**

| **Cause of death** | **n/N** | **Unadjusted** | |  | **Basic adjustment** | |  | **Extended adjustment** | |
| --- | --- | --- | --- | --- | --- | --- | --- | --- | --- |
|  |  | **HR (95% CI)** | **pint** |  | **HR (95% CI)** | **pint** |  | **HR (95% CI)** | **pint** |
| **All causes** |  |  |  |  |  |  |  |  |  |
| All participants |  |  |  |  |  |  |  |  |  |
| CKD-EPI 2021 | 2717/9691 | 2.44 (2.36-2.52)* | 0.59 |  | 1.26 (1.20-1.32)* | 0.28 |  | 1.28 (1.22-1.35)* | 0.38 |
| EKFC 2023 |  | 2.72 (2.62-2.81)* | 0.41 |  | 1.30 (1.23-1.37)* | 0.13 |  | 1.33 (1.25-1.40)* | 0.18 |
| Blacks |  |  |  |  |  |  |  |  |  |
| CKD-EPI 2021 | 455/1760 | 2.22 (2.05-2.40)* | … |  | 1.28 (1.14-1.43)* | … |  | 1.31 (1.17-1.47)* | … |
| EKFC 2023 |  | 2.43 (2.24-2.64)* | … |  | 1.30 (1.14-1.48)* | … |  | 1.34 (1.18-1.52)* | … |
| Non-Blacks |  |  |  |  |  |  |  |  |  |
| CKD-EPI 2021 | 2262/7931 | 2.54 (2.45-2.64)* | … |  | 1.23 (1.16-1.30)* | … |  | 1.25 (1.19-1.33)* | … |
| EKFC 2023 |  | 2.82 (2.72-2.93)* | … |  | 1.26 (1.18-1.35)* | … |  | 1.30 (1.21-1.38)* | … |
| **Cardiovascular** |  |  |  |  |  |  |  |  |  |
| All participants |  |  |  |  |  |  |  |  |  |
| CKD-EPI 2021 | 874/9691 | 2.65 (2.50-2.80)* | 0.72 |  | 1.36 (1.25-1.48)* | 0.95 |  | 1.38 (1.27-1.51)* | 0.80 |
| EKFC 2023 |  | 2.98 (2.81-3.17)* | 0.97 |  | 1.42 (1.29-1.56)* | 0.75 |  | 1.45 (1.32-1.60)* | 0.90 |
| Blacks |  |  |  |  |  |  |  |  |  |
| CKD-EPI 2021 | 143/1760 | 2.58 (2.24-2.97)* | … |  | 1.62 (1.33-1.97)* | … |  | 1.66 (1.37-2.03)* | … |
| EKFC 2023 |  | 2.86 (2.47-3.31)* | … |  | 1.71 (1.38-2.13)* | … |  | 1.77 (1.42-2.19)* | … |
| Non-Blacks |  |  |  |  |  |  |  |  |  |
| CKD-EPI 2021 | 731/7931 | 2.73 (2.56-2.90)* | … |  | 1.28 (1.16-1.42)* | … |  | 1.31 (1.19-1.44)* | … |
| EKFC 2023 |  | 3.07 (2.87-3.27)* | … |  | 1.32 (1.18-1.48)* | … |  | 1.35 (1.21-1.52)* | … |
| **Renal** |  |  |  |  |  |  |  |  |  |
| All participants |  |  |  |  |  |  |  |  |  |
| CKD-EPI 2021 | 66/9691 | 3.36 (2.76-4.08)* | 0.26 |  | 2.31 (1.77-3.01)* | 0.15 |  | 2.33 (1.79-3.03)* | 0.13 |
| EKFC 2023 |  | 3.70 (3.00-4.56)* | 0.17 |  | 2.53 (1.88-3.41)* | 0.12 |  | 2.56 (1.90-3.43)* | 0.10 |
| Blacks |  |  |  |  |  |  |  |  |  |
| CKD-EPI 2021 | 17/1760 | 2.81 (1.87-4.24)* | … |  | 1.78 (1.00-3.16) | … |  | 1.79 (1.01-3.15) | … |
| EKFC 2023 |  | 3.07 (1.99-4.73)* | … |  | 1.87 (0.99-3.52) | … |  | 1.87 (1.00-3.51) | … |
| Non-blacks |  |  |  |  |  |  |  |  |  |
| CKD-EPI 2021 | 49/7931 | 3.61 (2.87-4.53)* | … |  | 2.39 (1.74-3.28)* | … |  | 2.42 (1.77-3.31)* | … |
| EKFC 2023 |  | 4.00 (3.13-5.11)* | … |  | 2.66 (1.86-3.79)* | … |  | 2.69 (1.90-3.82)* | … |

Cardiovascular mortality includes ICD10 codes I00–I09, I11, I13, I20–I51, I60-I69 and renal mortality N00–N07, N17–N19, N25–N27. n/N indicates the number of deaths/ participants at risk. Hazard ratios (HR) are given with 95% confidence interval (CI) and express the risk per 1‑SD decrement in eGFR. Basic adjustment accounts for sex, age, body mass index and mean arterial pressure and extended adjustment additionally for smoking, educational attainment and the poverty index. pint is the race-by-eGFR interaction term. An ellipsis indicates not applicable. The eGFR equations are listed in table 1 (p 2) for CKD-EPI 2021 and in table 2 (p 3) for EKFC 2023. Significance of the HRs: * p≤0.0001.

*Table 9:*

**Association of mortality with eGFR derived from serum cystatin C in NHANES**

| **Cause of death** | **n/N** | **Unadjusted** | |  | **Partially adjusted** | |  | **Fully adjusted** | |  |
| --- | --- | --- | --- | --- | --- | --- | --- | --- | --- | --- |
|  |  | **HR (95% CI)** | **pint** |  | **HR (95% CI)** | **pint** |  | **HR (95% CI)** | **pint** | |
| **All causes** |  |  |  |  |  |  |  |  |  | |
| All participants |  |  |  |  |  |  |  |  |  | |
| CKD-EPI 2021 | 2717/9691 | 2.86 (2.77-2.96) | <0.0001 |  | 1.64 (1.56-1.73) | 0.46 |  | 1.59 (1.51-1.67) | 0.81 | |
| EKFC 2023 |  | 2.94 (2.86-3.03) | <0.0001 |  | 1.79 (1.70-1.89) | 0.29 |  | 1.73 (1.64-1.83) | 0.70 | |
| Blacks |  |  |  |  |  |  |  |  |  | |
| CKD-EPI 2021 | 455/1760 | 2.47 (2.30-2.65) | … |  | 1.72 (1.55-1.91) | … |  | 1.70 (1.52-1.89) | … | |
| EKFC 2023 |  | 2.51 (2.36-2.68) | … |  | 1.82 (1.64-2.03) | … |  | 1.82 (1.63-2.03) | … | |
| Non-Blacks |  |  |  |  |  |  |  |  |  | |
| CKD-EPI 2021 | 2262/7931 | 2.99 (2.88-3.10) | … |  | 1.63 (1.54-1.72) | … |  | 1.56 (1.48-1.66) | … | |
| EKFC 2023 |  | 3.09 (2.99-3.20) | … |  | 1.78 (1.67-1.89) | … |  | 1.70 (1.60-1.82) | … | |
| **Cardiovascular** |  |  |  |  |  |  |  |  |  | |
| All participants |  |  |  |  |  |  |  |  |  | |
| CKD-EPI 2021 | 874/9691 | 3.09 (2.93-3.27) | 0.010 |  | 1.72 (1.58-1.88) | 0.94 |  | 1.66 (1.53-1.82) | 0.75 | |
| EKFC 2023 |  | 3.15 (3.00-3.32) | 0.0010 |  | 1.88 (1.71-2.06) | 0.69 |  | 1.82 (1.65-2.00) | 0.91 | |
| Blacks |  |  |  |  |  |  |  |  |  | |
| CKD-EPI 2021 | 143/1760 | 2.74 (2.43-3.10) | … |  | 2.02 (1.70-2.39) | … |  | 2.02 (1.69-2.42) | … | |
| EKFC 2023 |  | 2.74 (2.46-3.06) | … |  | 2.13 (1.80-2.52) | … |  | 2.16 (1.82-2.58) | … | |
| Non-Blacks |  |  |  |  |  |  |  |  |  | |
| CKD-EPI 2021 | 731/7931 | 3.21 (3.01-3.42) | … |  | 1.65 (1.49-1.82) | … |  | 1.59 (1.44-1.75) | … | |
| EKFC 2023 |  | 3.30 (3.11-3.50) | … |  | 1.79 (1.61-2.00) | … |  | 1.72 (1.54-1.92) | … | |
| **Renal** |  |  |  |  |  |  |  |  |  |  |
| All participants |  |  |  |  |  |  |  |  |  |  |
| CKD–EPI 2021 | 66/9691 | 4.16 (3.42-5.06) | 0.081 |  | 3.23 (2.49-4.18) | 0.26 |  | 3.22 (2.47-4.18) | 0.25 |  |
| EKFC 2023 |  | 3.94 (3.32-4.69) | 0.046 |  | 3.29 (2.59-4.17) | 0.18 |  | 3.29 (2.58-4.20) | 0.18 |  |
| Blacks |  |  |  |  |  |  |  |  |  |  |
| CKD–EPI 2021 | 17/1760 | 3.39 (2.42-4.74) | … |  | 2.78 (1.78-4.36) | … |  | 2.77 (1.74-4.42) | … |  |
| EKFC 2023 |  | 3.18 (2.34-4.31) | … |  | 2.72 (1.76-4.22) | … |  | 2.73 (1.73-4.31) | … |  |
| Non-Blacks |  |  |  |  |  |  |  |  |  |  |
| CKD–EPI 2021 | 49/7931 | 4.57 (3.59-5.81) | … |  | 3.40 (2.49-4.66) | … |  | 3.41 (2.48-4.68) | … |  |
| EKFC 2023 |  | 4.36 (3.53-5.39) | … |  | 3.53 (2.65-4.69) | … |  | 3.55 (2.65-4.74) | … |  |

Cardiovascular mortality includes ICD10 codes I00–I09, I11, I13, I20–I51, I60-I69 and renal mortality N00–N07, N17–N19, N25–N27. n/N indicates the number of deaths/ participants at risk. Hazard ratios (HR) are given with 95% confidence interval (CI) and express the risk per 1‑SD decrement in eGFR. Basic adjustment accounts for sex, age, body mass index and mean arterial pressure and extended adjustment additionally for smoking, educational attainment and the poverty index. pint is the race-by-eGFR interaction term. An ellipsis indicates not applicable. The eGFR equations are listed in table 1 (p 2) for CKD-EPI 2021 and in table 2 (p 3) for EKFC 2023. All HRs are significant (p<0.0001).

*Table 10:*

**Association of mortality with eGFR derived from serum creatinine and cystatin C in NHANES**

| **Cause of death** | **n/N** | **Unadjusted** | |  | **Basic adjustment** | |  | **Extended adjustment** | |
| --- | --- | --- | --- | --- | --- | --- | --- | --- | --- |
|  |  | **HR (95% CI)** | **pint** |  | **HR (95% CI)** | **pint** |  | **HR (95% CI)** | **pint** |
| **All causes** |  |  |  |  |  |  |  |  |  |
| All participants |  |  |  |  |  |  |  |  |  |
| CKD-EPI 2021 | 2717/9691 | 2.62 (2.54-2.70) | <0.0001 |  | 1.49 (1.42-1.56) | 0.36 |  | 1.47 (1.40-1.54) | 0.59 |
| EKFC 2023 |  | 2.87 (2.78-2.95) | <0.0001 |  | 1.60 (1.51-1.69) | 0.21 |  | 1.59 (1.50-1.68) | 0.41 |
| Blacks |  |  |  |  |  |  |  |  |  |
| CKD-EPI 2021 | 455/1760 | 2.31 (2.15-2.47) | … |  | 1.53 (1.37-1.69) | … |  | 1.52 (1.37-1.69) | … |
| EKFC 2023 |  | 2.50 (2.34-2.68) | … |  | 1.63 (1.44-1.84) | … |  | 1.64 (1.45-1.85) | … |
| Non-Blacks |  |  |  |  |  |  |  |  |  |
| CKD-EPI 2021 | 2262/7931 | 2.72 (2.63-2.82) | … |  | 1.47 (1.39-1.55) | … |  | 1.44 (1.37-1.52) | … |
| EKFC 2023 |  | 2.99 (2.89-3.09) | … |  | 1.57 (1.47-1.67) | … |  | 1.55 (1.45-1.65) | … |
| **Cardiovascular** |  |  |  |  |  |  |  |  |  |
| All participants |  |  |  |  |  |  |  |  |  |
| CKD-EPI 2021 | 874/9691 | 2.82 (2.68-2.97) | 0.048 |  | 1.58 (1.45-1.71) | 0.96 |  | 1.56 (1.43-1.69) | 0.72 |
| EKFC 2023 |  | 3.09 (2.93-3.25) | 0.021 |  | 1.72 (1.56-1.90) | 0.75 |  | 1.70 (1.55-1.88) | 0.97 |
| Blacks |  |  |  |  |  |  |  |  |  |
| CKD-EPI 2021 | 143/1760 | 2.59 (2.30-2.92) | … |  | 1.85 (1.55-2.20) | … |  | 1.86 (1.56-2.22) | … |
| EKFC 2023 |  | 2.80 (2.49-3.15) | … |  | 2.03 (1.68-2.45) | … |  | 2.07 (1.71-2.51) | … |
| Non-Blacks |  |  |  |  |  |  |  |  |  |
| CKD-EPI 2021 | 731/7931 | 2.90 (2.74-3.08) | … |  | 1.50 (1.37-1.65) | … |  | 1.48 (1.35-1.62) | … |
| EKFC 2023 |  | 3.20 (3.01-3.39) | … |  | 1.61 (1.44-1.80) | … |  | 1.59 (1.42-1.78) | … |
| **Renal** |  |  |  |  |  |  |  |  |  |
| All participants |  |  |  |  |  |  |  |  |  |
| CKD-EPI 2021 | 66/9691 | 3.64 (3.05-4.35) | 0.13 |  | 2.82 (2.22-3.58) | 0.27 |  | 2.82 (2.21-3.59) | 0.26 |
| EKFC 2023 |  | 3.79 (3.18-4.53) | 0.094 |  | 3.06 (2.38-3.93) | 0.18 |  | 3.07 (2.38-3.96) | 0.17 |
| Blacks |  |  |  |  |  |  |  |  |  |
| CKD-EPI 2021 | 17/1760 | 3.09 (2.21-4.31) | … |  | 2.45 (1.53-3.90) | … |  | 2.43 (1.51-3.92) | … |
| EKFC 2023 |  | 3.15 (2.25-4.42) | … |  | 2.49 (1.49-4.17) | … |  | 2.49 (1.47-4.23) | … |
| Non-Blacks |  |  |  |  |  |  |  |  |  |
| CKD-EPI 2021 | 49/7931 | 3.91 (3.17-4.84) | … |  | 2.90 (2.18-3.86) | … |  | 2.90 (2.18-3.87) | … |
| EKFC 2023 |  | 4.12 (3.33-5.10) | … |  | 3.22 (2.39-4.34) | … |  | 3.24 (2.40-4.37) | … |

Cardiovascular mortality includes ICD10 codes I00–I09, I11, I13, I20–I51, I60-I69 and renal mortality N00–N07, N17–N19, N25–N27. n/N indicates the number of deaths/ participants at risk. Hazard ratios (HR) are given with 95% confidence interval (CI) and express the risk per 1‑SD decrement in eGFR. Basic adjustment accounts for sex, age, body mass index and mean arterial pressure and extended adjustment additionally for smoking, educational attainment and the poverty index. pint is the race-by-eGFR interaction term. An ellipsis indicates not applicable. The eGFR equations are listed in table 1 (p 2) for CKD-EPI 2021 and in table 2 (p 3) for EKFC 2023. All HRs are significant (p<0.0007).

***Table 11:***

**AUC for mortality in relation to race-free eGFR equations in NHANES**

| **Endpoint   eGFR equation   Consortium** |  | **Blacks** | |  | **Non-Blacks** | |
| --- | --- | --- | --- | --- | --- | --- |
|  |  | **AUC (95%CI)** | **p value** |  | **AUC (95% CI)** | **p value** |
| **All-cause mortality** |  |  |  |  |  |  |
| Base model |  | 0.837 (0.818–0.856) |  |  | 0.857 (0.851–0.864) |  |
| eGFRcr |  |  |  |  |  |  |
| CKD-EPI 2021 |  | 0.841 (0.822–0.860) | 0.051 |  | 0.859 (0.852–0.865) | 0.0007 |
| EKFC 2023 |  | 0.840 (0.820–0.860) | 0.23 |  | 0.859 (0.852–0.865) | 0.0024 |
| eGFRcys |  |  |  |  |  |  |
| CKD-EPI 2021 |  | 0.849 (0.830–0.868) | 0.0007 |  | 0.864 (0.857–0.870) | <0.0001 |
| EKFC 2023 |  | 0.849 (0.831–0.867) | <0.0001 |  | 0.864 (0.857–0.870) | <0.0001 |
| eGFRcr-cys |  |  |  |  |  |  |
| CKD-EPI 2021 |  | 0.846 (0.827–0.864) | 0.0033 |  | 0.862 (0.855–0.868) | <0.0001 |
| EKFC 2023 |  | 0.844 (0.824–0.864) | 0.029 |  | 0.861 (0.855–0.868) | <0.0001 |
| **Cardiovascular mortality** |  |  |  |  |  |  |
| Base model |  | 0.868 (0.842–0.894) |  |  | 0.883 (0.872–0.895) |  |
| eGFRcr |  |  |  |  |  |  |
| CKD-EPI 2021 |  | 0.884 (0.860–0.907) | 0.036 |  | 0.885 (0.873–0.896) | 0.091 |
| EKFC 2023 |  | 0.884 (0.860–0.907) | 0.038 |  | 0.885 (0.873–0.896) | 0.13 |
| eGFRcys |  |  |  |  |  |  |
| CKD-EPI 2021 |  | 0.891 (0.869–0.912) | 0.0044 |  | 0.888 (0.876–0.899) | 0.0006 |
| EKFC 2023 |  | 0.888 (0.867–0.910) | 0.0091 |  | 0.888 (0.877–0.900) | 0.0001 |
| eGFRcr-cys |  |  |  |  |  |  |
| CKD-EPI 2021 |  | 0.890 (0.867–0.912) | 0.0057 |  | 0.887 (0.875–0.898) | 0.0060 |
| EKFC 2023 |  | 0.889 (0.866–0.911) | 0.010 |  | 0.887 (0.875–0.898) | 0.0032 |

AUC indicates the area under the curve and eGFRcr/eGFRcys/eGFRcr-cys eGFR derived from serum creatinine/cystatin C/both markers. The base model includes sex, age, body mass index, mean arterial pressure, smoking, educational attainment and the poverty index. The poverty index is the ratio of family income to poverty as defined in each NHANES survey year by the Department of Health and Human Services, higher values indicating greater affluence. p values are for the significance of the AUC increase. An ellipsis indicates not applicable. The eGFR equations are listed in table 1 (p 2) for CKD-EPI 2021 and in table 2 (p 3) for EKFC 2023.


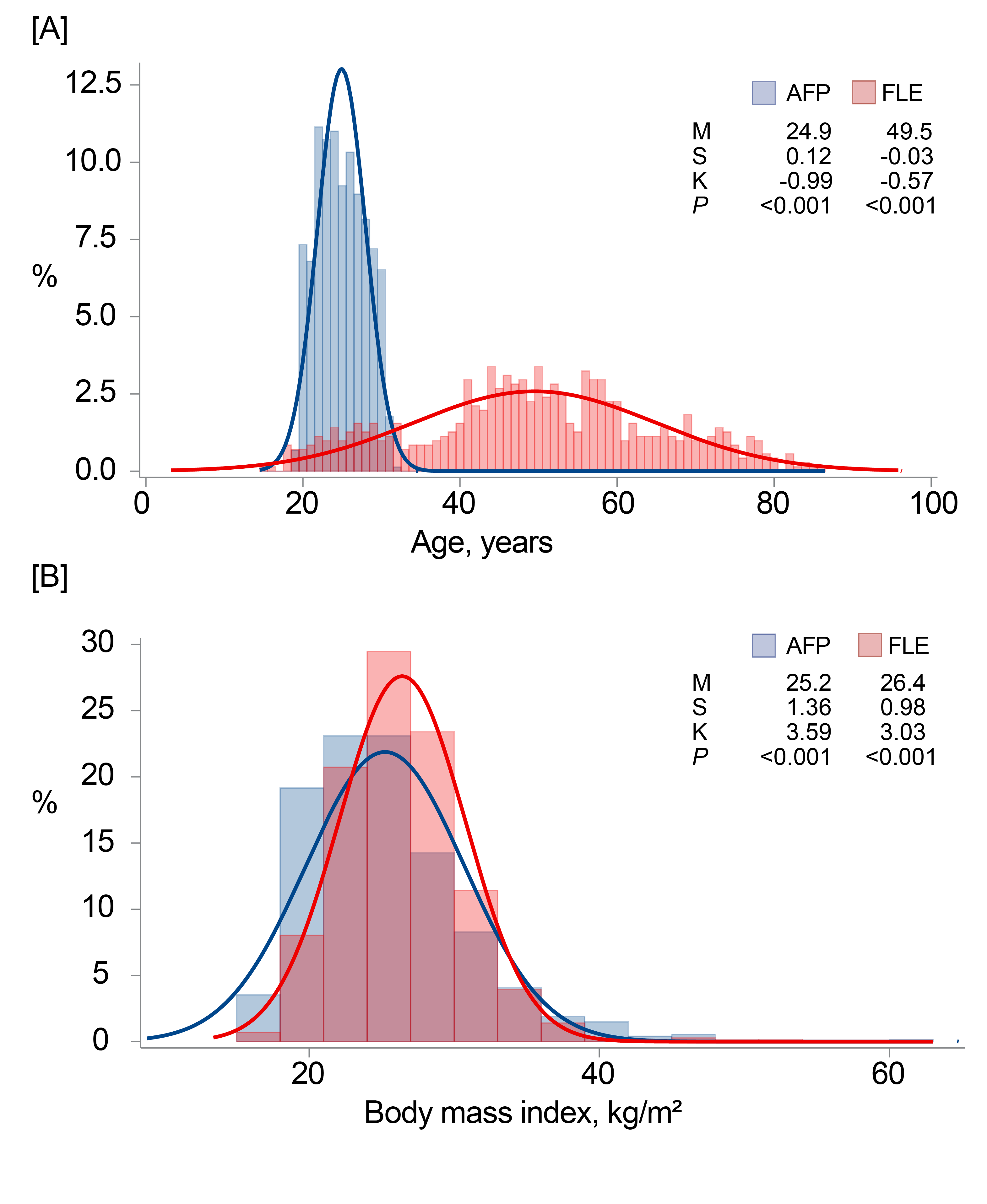


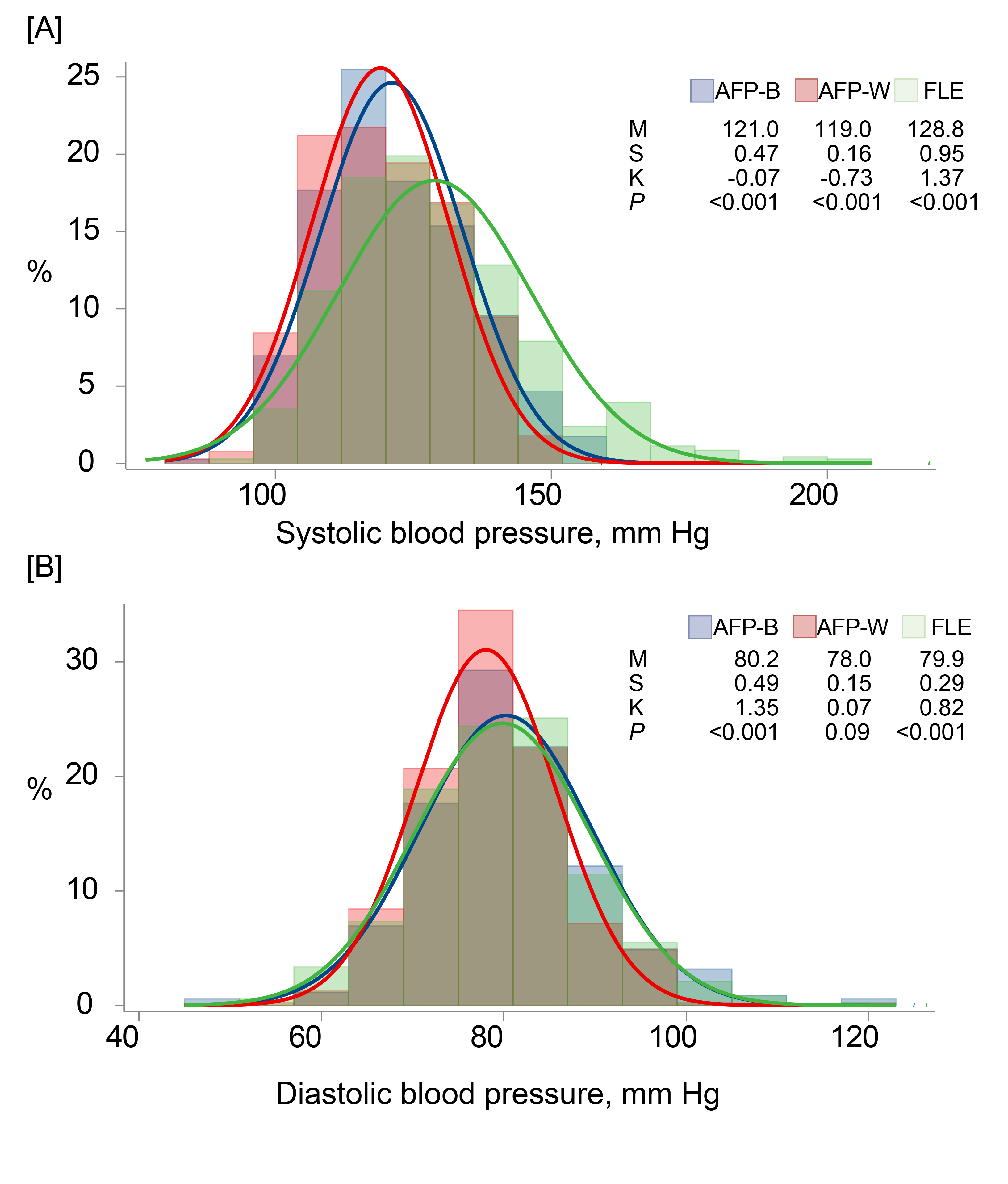


***Figure 1:***

**Distributions of age (A) and body mass index (B) in 721 Black and White African-PREDICT and 709 White FLEMENGHO participants**
AFP and FLE indicate African-PREDICT and FLEMENGHO, respectively. The solid lines represent the normal distribution. p-values are for departure of the actually observed distribution from normality according to the Shapiro-Wilk statistic. M indicates the arithmetic mean. Skewness (S) and kurtosis (K) are computed as the third and fourth moment about the mean divided by the cube of the standard deviation.


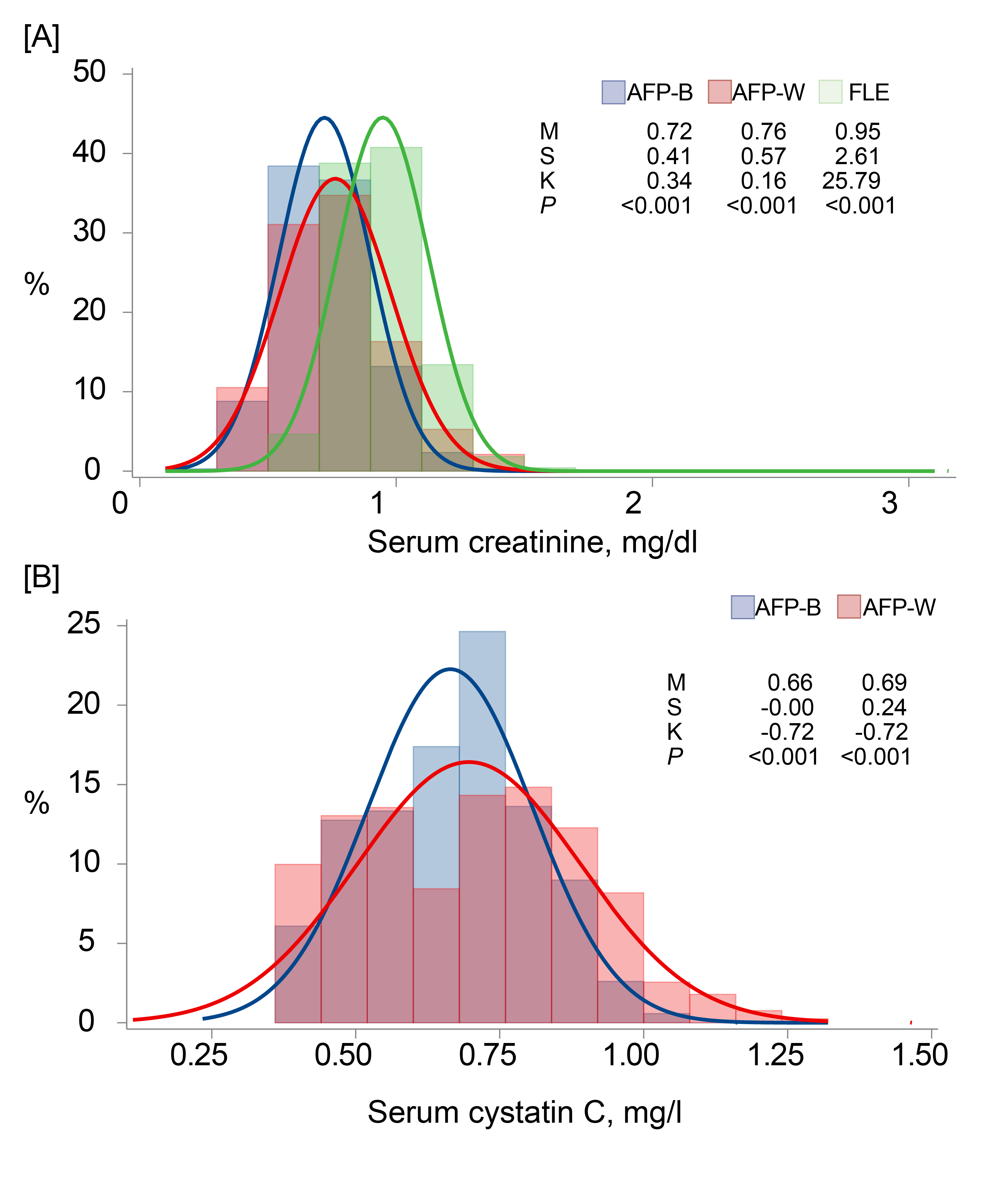


***Figure 2:***

**Distributions of systolic (A) and diastolic (B) blood pressure in Black and White African-PREDICT and White FLEMENGHO participants**
AFP and FLE indicate African-PREDICT and FLEMENGHO, respectively. The solid lines represent the normal distribution. p-values are for departure of the actually observed distribution from normality according to the Shapiro-Wilk statistic. M indicates the arithmetic mean. Skewness (S) and kurtosis (K) are computed as the third and fourth moment about the mean divided by the cube of the standard deviation.


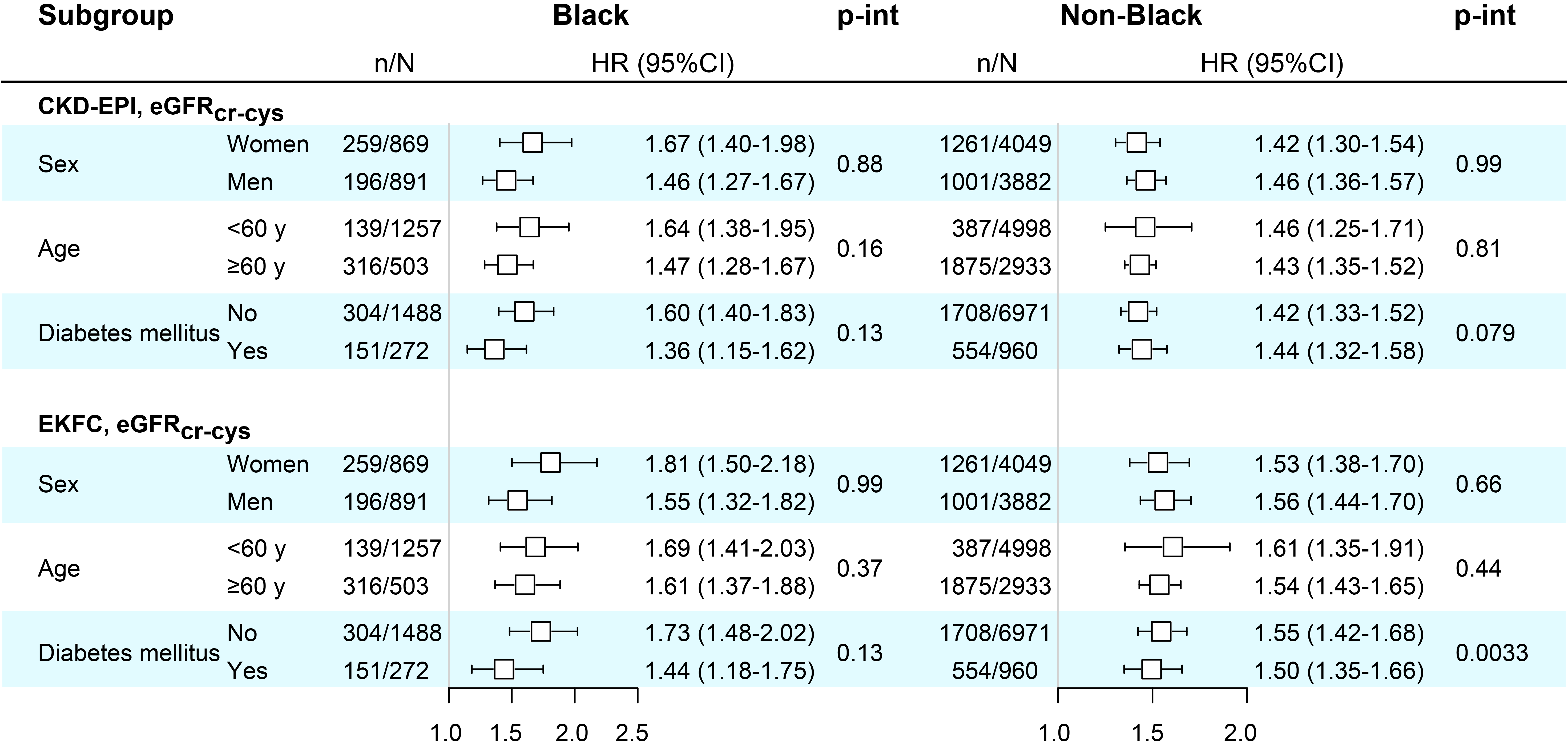


***Figure 3:***

**Distributions of serum creatinine (A) and serum cystatin C (B) concentration in Black and White African-PREDICT and White FLEMENGHO participants**
AFP and FLE indicate African-PREDICT and FLEMENGHO, respectively. Serum cystatin C was only measured in African-PREDICT. The solid lines represent the normal distribution. p-values are for departure of the actually observed distribution from normality according to the Shapiro-Wilk statistic. M indicates the arithmetic mean. Skewness (S) and kurtosis (K) are computed as the third and fourth moment about the mean divided by the cube of the standard deviation. Conversion factors: creatinine from mg/dL to μmol/L, multiply by 88.42; cystatin C from mg/L to nmol/L, multiply by 74.9.


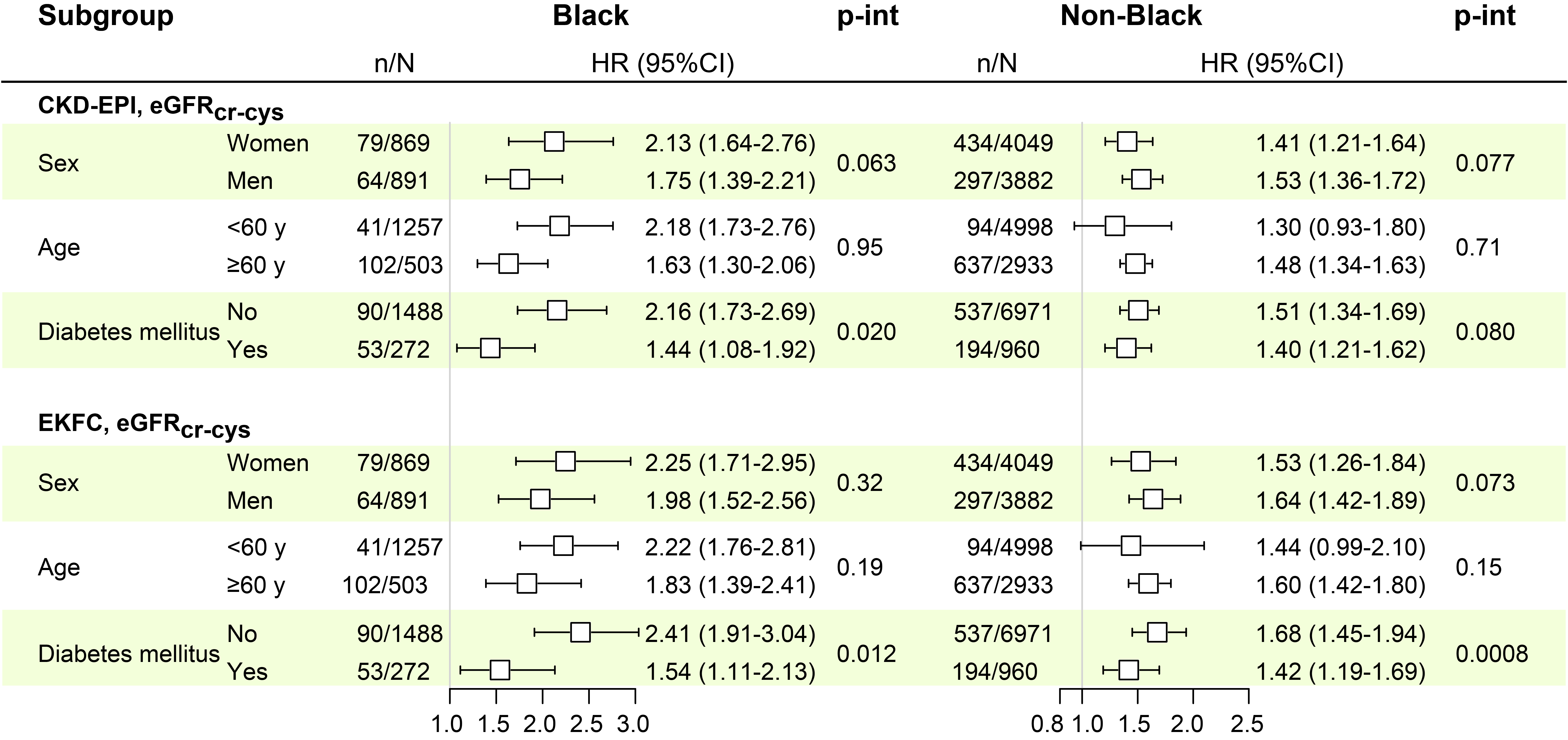


***Figure 4:***

**Subgroup analysis of the association of all-cause mortality with eGFR derived from both serum creatinine and cystatin C in NHANES participants**
n/N(B) and n/N(W) refer to the number of deaths/participants at risk in Blacks and non-Blacks. Hazard ratios, given with 95% confidence interval, express the risk associated with a 1-SD decrement in eGFR. Models are adjusted for sex (not applicable for the categorisation by sex), age, body mass index, mean arterial pressure, smoking, educational attainment and the poverty index. The poverty index is the ratio of family income to poverty as defined in each NHANES survey year by the Department of Health and Human Services, higher values indicating greater affluence. p-int is the significance of the subgroup-by-eGFR interaction term.

***Figure 5:***

**Subgroup analysis of the association of cardiovascular mortality with eGFR derived from both serum creatinine and cystatin C in NHANES participants**
n/N refers to the number of deaths/participants at risk in Blacks and non-Blacks. Hazard ratios, given with 95% confidence interval, express the risk associated with a 1-SD decrement in eGFR. Models are adjusted for sex (not applicable for the categorisation by sex), age, body mass index, mean arterial pressure, smoking, educational attainment and the poverty index. The poverty index is the ratio of family income to poverty as defined in each NHANES survey year by the Department of Health and Human Services, higher values indicating greater affluence. p-int is the significance of the subgroup-by-eGFR interaction term.
